# Kinetics of Thermal Denaturation and Aggregation of Bovine Serum Albumin

**DOI:** 10.1371/journal.pone.0153495

**Published:** 2016-04-21

**Authors:** Vera A. Borzova, Kira A. Markossian, Natalia A. Chebotareva, Sergey Yu. Kleymenov, Nikolay B. Poliansky, Konstantin O. Muranov, Vita A. Stein-Margolina, Vladimir V. Shubin, Denis I. Markov, Boris I. Kurganov

**Affiliations:** 1 Department of Structural Biochemistry of Proteins, Bach Institute of Biochemistry, Research Center of Biotechnology of the Russian Academy of Sciences, Moscow, Russia; 2 Department of Biophysics, Kol’tsov Institute of Developmental Biology, Russian Academy of Sciences, Moscow, Russia; 3 Department of Chemical and Biological Processes Kinetics, Emanuel Institute of Biochemical Physics, Russian Academy of Sciences, Moscow, Russia; 4 Department of Biochemistry of Chloroplast, Bach Institute of Biochemistry, Research Center of Biotechnology of the Russian Academy of Sciences, Moscow, Russia; Russian Academy of Sciences, Institute for Biological Instrumentation, RUSSIAN FEDERATION

## Abstract

Thermal aggregation of bovine serum albumin (BSA) has been studied using dynamic light scattering, asymmetric flow field-flow fractionation and analytical ultracentrifugation. The studies were carried out at fixed temperatures (60°C, 65°C, 70°C and 80°C) in 0.1 M phosphate buffer, pH 7.0, at BSA concentration of 1 mg/ml. Thermal denaturation of the protein was studied by differential scanning calorimetry. Analysis of the experimental data shows that at 65°C the stage of protein unfolding and individual stages of protein aggregation are markedly separated in time. This circumstance allowed us to propose the following mechanism of thermal aggregation of BSA. Protein unfolding results in the formation of two forms of the non-native protein with different propensity to aggregation. One of the forms (highly reactive unfolded form, U_hr_) is characterized by a high rate of aggregation. Aggregation of U_hr_ leads to the formation of primary aggregates with the hydrodynamic radius (*R*_h,1_) of 10.3 nm. The second form (low reactive unfolded form, U_lr_) participates in the aggregation process by its attachment to the primary aggregates produced by the U_hr_ form and possesses ability for self-aggregation with formation of stable small-sized aggregates (A_st_). At complete exhaustion of U_lr_, secondary aggregates with the hydrodynamic radius (*R*_h,2_) of 12.8 nm are formed. At 60°C the rates of unfolding and aggregation are commensurate, at 70°C the rates of formation of the primary and secondary aggregates are commensurate, at 80°C the registration of the initial stages of aggregation is complicated by formation of large-sized aggregates.

## Introduction

Stability of most proteins is dependent on certain conditions, including temperature, pH and ionic strength. Native proteins can unfold in the presence of denaturing agents as well as a result of exposure to high temperatures. Unfolded proteins may interact by the exposed hydrophobic sequences with formation of amorphous or amyloid aggregates [1–6].

Thermal aggregation of proteins includes the stage of protein unfolding followed by aggregation of unfolded protein molecules. The kinetic regime of the general process of thermal aggregation of proteins is determined by the relationship between the rates of unfolding and aggregation stages. If the rate of the aggregation stage is significantly higher than the rate of the unfolding stage, the rate-limiting stage of the general process of aggregation will be that of protein unfolding. To prove the fulfillment of this kinetic regime for glyceraldehyde-3-phoshate dehydrogenase (GAPDH) from rabbit skeletal muscles, we studied aggregation of this protein under conditions when the temperature was increased at a constant rate [7]. Aggregation was registered by measurement of the increment in the light scattering intensity. Denaturation of GAPDH was controlled by differential scanning calorimetry (DSC). Linear character of the dependence of light scattering intensity on the portion of the denatured protein was interpreted as an evidence for the fulfillment of the kinetic regime where protein denaturation is the rate-limiting stage.

To obtain more substantiated information on the kinetic regime of thermal aggregation of proteins, experiments should be performed as follows. First of all, it is required to use the direct methods of determination of aggregated protein concentration. A further comparison of the portions of denatured and aggregation protein allows us to make a conclusion about the nature of the rate-limiting stage. In the present work we used this approach to study the kinetics of thermal aggregation of bovine serum albumin (BSA).

BSA, a water-soluble monomeric protein with molecular mass of 66.4 kDa [8] is composed of three homologous domains, formed by six α-helices [9]. Polypeptide chain of BSA, consisting of 583 amino acid residues [10], contains only two tryptophan residues [11,12]. Two tryptophans in BSA molecule are embedded in two different domains: Trp-134, located in proximity of the protein surface, but buried in a hydrophobic pocket of domain I, and Trp-214, located in an internal part of domain II [12].

BSA has 17 intrachain disulphide bridges and one sulfhydryl group (Cys-34) [8,13]. The disulphide bonds give some rigidity to each sub-domain, but allow for a significant modification in the shape and size of the protein under different external conditions [8,13,14]. At neutral pH the disulphide bridges are buried in the protein molecule and are not exposed to the solvent [15]. A unique free cystein (Cys-34) is located in domain I in a hydrophobic pocket of the BSA molecule [16].

BSA undergoes reversible conformational isomerization depending on pH, transforming from the compact N form (at neutral pH) to the less compact F form (such as at pH 4) [16,17]. Under heating the compact native form of BSA becomes more flexible and more reactive [18,19], and Tyr and Trp residues are exposed to a more polar environment [20]. The thermal behavior of BSA is dependent on pH of incubation medium. On the basis of DSC experiments it has been shown that at pH values 6.0–8.5 the denaturation process of the BSA is a sum of two independent one-step transitions [21,22]. It has been reported that at pH 7 the N-terminal domain I of the BSA molecule unfolds and the domains II and III melt together at higher temperatures [23]. Such behavior of BSA under heating is determined by the multidomain structure of BSA [24].

Unfolding of BSA induced by heating of aqueous solutions of the protein causes its aggregation [19,25–27]. The rate of heat-induced aggregation of BSA depends on temperature, pH values, protein concentration, incubation time and salt concentration [28]. All these factors affect the size of the aggregates. According to the dynamic light scattering (DLS) data, increase in temperature from 30°C to 60°C at pH 7.0 resulted in a slight growth in the hydrodynamic radius (*R*_h_) of BSA molecules [20,29]. However, under heating above 65°C for 10 min an exponential increase in *R*_h_ was observed, evidencing the occurrence of aggregation of BSA. A rapid growth of the hydrodynamic radius of BSA aggregates at the early stages (over the initial 30 min) was followed by a minor increase for time interval 30–180 min. The study of heat-induced aggregation of BSA by asymmetric flow field-flow fractionation (AF4) also allowed showing that the hydrodynamic diameter of BSA aggregates depends on concentration of the protein, duration of incubation and the ionic strength of the solvent [30].

The data available indicate that the mechanism of heat-induced BSA aggregation proceeds via two different pathways: formation of relatively small “soluble”aggregates by the conversion of α-helix to β-aggregated structures as a result of electrostatic interactions or formation of aggregates of larger size via hydrophobic interactions [31,32]. According to Honda et al. [26], two thermal aggregation processes are observed at relatively low temperatures and low concentrations of BSA. The first proceeds by an inter-monomer aggregation mechanism, and the second one by inter-aggregates aggregation. In the first process, monomers of BSA aggregate, their particle size increases and almost all monomers merge into an aggregate. The first process proceeds in the regime of diffusion-limited cluster-cluster aggregation (DLCA). In the second process the reaction of inter-aggregates takes place and particle size increases rapidly. When the particle size reaches a plateau, the particles no longer aggregate, because the concentration of the remaining monomeric BSA is too low to aggregate further. The particle size in this plateau region depends on the temperature.

In recent work by Sahin et al. [33] thermal aggregation of BSA was studied using SEC-HPLC in temperature interval from 50°C to 70°C. Results showed that BSA aggregated irreversibly through both sequential monomer addition and aggregate-aggregate interactions. Aggregate-aggregate interactions were significant above 63–65°C, particularly at later stages of aggregation when sequential monomer addition seemed to cease.

Holm et al. [34] showed that amyloid aggregates of BSA were formed in a few minutes after the start of incubation of the protein at neutral pH at elevated temperatures. The reaction proceeded without a lag phase and was not accelerated by seeding. Amyloid aggregates can be cytotoxic or they can be functional and healthful in some cases. BSA amyloid aggregates are non-cytotoxic [34], they do not cause amyloid diseases, and they may have some practical application.

Analysis of literature data on thermal aggregation of BSA shows that additional kinetic investigations with the involvement of different physical and physico-chemical methods should be carry out to build a complete picture of pathways of BSA aggregation. Particular attention has to be given to the comparison of the portions of denatured and aggregated protein and possibility of the change in the rate-limiting stage of the overall aggregation process at varying the temperature of the aggregation experiment.

Commercial preparations of BSA are easily available. Therefore BSA is a suitable subject for construction of test systems designed for screening of agents possessing anti-aggregation activity. Shah et al. [35] used a test system based on thermal aggregation of BSA for the study of anti-aggregation activity of arginine. Several works are devoted to using a test system based on dithiothreitol-induced aggregation of BSA for testing chaperone-like activity of chaperones of a proteinous nature and chemical chaperones [36–38]. To elucidate the mechanism of anti-aggregation activity of chaperones and estimate quantitatively their protective efficiency, an investigation of the mechanism of protein substrate aggregation is needed.

In the present work we studied the kinetics of thermal aggregation of BSA in the temperature interval from 60°C to 80°C using DLS and AF4. To control unfolding of BSA, DSC was used. Morphology of BSA aggregates was studied using transmission electron microscopy (TEM). The relationship between non-aggregated and aggregated forms in the preparation of BSA preincubated at 60°C was also characterized using analytical ultracentrifugation (AUC). To isolate the fraction of non-aggregated unfolded form of BSA, size-exclusion chromatography (SEC) was applied. Based on the analysis of the relationship between the portion of the aggregated protein (γ_agg_) and portion of the denatured protein and the relationship between the light scattering intensity and γ_agg_, a model of thermal aggregation of BSA has been developed. The model involves formation of the primary aggregates with participation of highly reactive unfolded form of BSA, growth of the primary aggregates as a result of the attachment of low reactive unfolded form of BSA and further sticking of the newly-formed secondary aggregates with formation of polydisperse amorphous aggregates.

## Materials and Methods

### Materials

BSA (fatty acid depleted, catalogue no. A7638, 99+ % of purity), sodium phosphate monobasic, sodium phosphate dibasic and sodium chloride were purchased from Sigma—Aldrich and used without further purification. The fluorescent probes thioflavin T (ThT) and 8-anilinonaphthalene-1-sulfonic acid (ANS) were obtained from Sigma—Aldrich. All solutions for the experiments were prepared using deionized water obtained with Easy-Pure II RF system (Barnstead, USA). BSA samples were prepared by dissolving solid BSA in 0.1 M phosphate buffer solutions at pH 7.0. All experiments were performed with freshly prepared solutions of BSA. BSA concentration was determined spectrophotometrically at 280 nm using the absorption coefficient $A_{\text{cm}}^{1\%}$ of 6.58 [39].

### Size-Exclusion Chromatography

The protein samples were loaded onto the column (Sephacryl S100 HR) and separated into fractions at a flow rate of 2.5 ml/min (20°C). The column was pre-calibrated with the following proteins (Sigma—Aldrich): thyroglobulin (660 kDa), catalase (440 kDa), aldolase (158 kDa), BSA (67 kDa), γ-crystallin (20 kDa). The relative error for protein mass determination was 4%.

### Calorimetric Studies

Thermal denaturation of BSA in 0.1 M sodium phosphate buffer, pH 7.0, was studied by DSC using adiabatic scanning microcalorimeter DASM-4M (Institute of Biological Instruments, Russian Academy of Sciences, Pushchino, Russia) with 0.47 ml capillary platinum cells. All measurements were carried out at the rate of heating by 1°C/min in the temperature range from 42°C to 70°C and at constant pressure of 2.2 atm. The dependences of heat power on temperature were calculated using Origin software (MicroCal, Inc., USA). The capillary construction of calorimetric cells prevents the artifacts caused by protein precipitation, which are often observed in batch calorimetric cells as exothermic peaks. All measurements were repeated three times for each sample.

### Transmission Electron Microscopy

The aliquots of BSA were added to preheated 0.1 M Na-phosphate buffer, pH 7.0, to the final protein concentration of 1 mg/ml and were incubated at 65°C for 110 or 330 min. Samples were cooled to room temperature and dialyzed against deionized water at 4–8°C for 16 h. Aliquots of each dialyzed sample were diluted 100 times with deionized water.

Drops of the diluted and non-diluted BSA samples were placed onto carbon-coated copper grids. A drop of the tobacco mosaic virus solution was added for reference as an object with known diameter (15–18 nm). The excess of the samples was removed using a piece of filter paper. Grids containing non-diluted samples were additionally washed in deionized water.

Grids were stained with 2% (w/v) phosphotungstic acid, pH 7.2, (the excess was removed using a piece of filter paper) and were air-dried at room temperature. Images were obtained using JEOL-JEM100-CX transmission electron microscope operating at an accelerating voltage of 80 kV.

### Circular Dichroism Spectroscopy

CD spectra and absorption spectra of BSA solutions were recorded on Chirascan spectrometer (Applied Photophysics, UK) in the interval of wavelengths 185–320 nm, with 1.5-nm slit width and 0.5-nm step at 20°C. The concentration of the protein in all samples was about 0.1 mg/ml (0.01 M Na-phosphate buffer, pH 7.0). Optical path length was 0.1 cm. The exact protein concentration was determined using extinction coefficients of peptide bond at 205, 210 and 215 nm [40]. All measurements were repeated three times for each sample.

### Fluorescence Measurements

Fluorescence spectra were recorded on fluorescence spectrophotometer Cary Eclipse (Varian, Agilent Technologies, Inc., USA). BSA samples (1 mg/ml) were preincubated for 12 h at 60°C, 65°C, 70°C or 80°C. The fluorescence intensity of ThT solution and ThT incubated with preheated BSA (0.4 mg/ml) for 1 h at room temperature was measured with excitation at 450 nm (slit width 5 nm). The fluorescence intensity of ANS (final concentration 10 μM) incubated with BSA (0.1 mg/ml) for 1 h at room temperature was measured with excitation at 380 nm (slit width 5 nm).

### Zeta Potential Measurements

Zeta potential of BSA was measured using Photocor Compact-Z instrument (Photocor Instruments, Inc., USA). Laser with wavelength 654 nm was used as a light source. The measurements were conducted at electrical field voltage 5 V/cm and 23°C in cylindrical glass vials with disposable Au electrodes. The distance between electrodes was 0.4 cm. The scattered light was collected at a 20° angle. The experiment was repeated three times for each sample.

### Light Scattering Intensity Measurements

For light scattering measurements a commercial instrument Photocor Complex (Photocor Instruments, Inc., USA) was used. A He—Ne laser (Coherent, USA, Model 31–2082, 632.8 nm, 10 mW) was used as a light source. DynaLS software (Alango, Israel) was used for polydisperse analysis of DLS data. The diffusion coefficient *D* of the particles is directly related to the decay rate τ_c_ of the time-dependent correlation function for the light scattering intensity fluctuations:

$$D=1/2\tau_{c}k^{2}.$$ (1)

In this equation *k* is the wave number of the scattered light, *k* = (4π*n*/θ)sin(θ/2), where *n* is the refractive index of the solvent, λ is the wavelength of the incident light in vacuum and θ is the scattering angle. The mean hydrodynamic radius of the particles, *R*_h_, can be calculated according to Stokes—Einstein equation:

$$D=k_{B}T/6\pi \eta R_{h},$$ (2)

where *k*_B_ is Boltzmann’s constant, *T* is the absolute temperature and η is the dynamic viscosity of the solvent.

The kinetics of heat-induced aggregation of BSA was studied in 0.1 M Na-phosphate buffer, pH 7.0. The buffer was placed in a cylindrical cell with the internal diameter of 6.3 mm and preincubated for 5 min at a given temperature. Cells with stopper were used to avoid evaporation. The aggregation process was initiated by the addition of an aliquot of BSA solution to a preheated buffer to the final volume of 0.5 ml. When studying the kinetics of aggregation of BSA, the scattering light was collected at a 90° scattering angle.

In the case of diffusion-limited cluster-cluster aggregation (DLCA) the dependence of the hydrodynamic radius (*R*_h_) of protein aggregates on time follows the power law [41,42]:

$$R_{\text{h}}=R_{\text{h}}^{*}\;[1+K(t-t^{*})]{\;}^{1/d{\;}_{\text{f}}},\;(t>t^{*}),$$ (3)

where t* is the moment of time when the kinetic regime starts to fulfill, $R_{\text{h}}^{*}$ is the hydrodynamic radius at *t* = t*, *K* is the constant and *d*_f_ is the fractal dimension of aggregates (*d*_f_ = 1.8 for DLCA kinetic regime).

### Asymmetric Flow Field-Flow Fractionation with On-Line Multi-Angle Light Scattering (MALS), Ultraviolet (UV) and Refractive Index (RI) Detectors

Eclipse 3 separation system (Wyatt Technology Corporation, USA) based on an Agilent HPLC pump (Agilent Technologies, USA) was used for AF4 experiments. BSA sample in 0.1 M Na-phosphate buffer, pH 7.0, preheated at 60°C, 65°C, 70°C and 80°C and cooled to room temperature 23°C, was injected in the separation channel by Agilent autoinjection system (Agilent Technologies, USA). A 21.4 cm channel with a 350-μm channel spacer and ultrafiltration membrane made of regenerated cellulose with a 10-kDa molecular weight cut off (Wyatt Technology Corporation, USA) were used. The flow system was sequentially connected to UV detector (Agilent Technologies, USA), MALS detector (DAWN HELEOS II, Wyatt Technology Corporation, USA) and RI detector (Optilab T-rEX, Wyatt Technology Corporation, USA). The elution was performed with 0.1 M phosphate buffer, pH 7.0. The data from the detectors were processed in ASTRA software, version 5.3.4 (Wyatt Technology Corporation, USA) to yield the final profiles. The experiment was carried out at room temperature (23°C).

### Analytical Ultracentrifugation

Sedimentation velocity experiments were carried out at 25°C in a Model E analytical ultracentrifuge (Beckman), equipped with absorbance optics, a photoelectric scanner, a monochromator and an on-line computer. A four-hole An-F Ti rotor and 12 mm double sector cells were used. The sedimentation profiles of BSA (0.1 M Na-phosphate buffer, pH 7.0, containing 10 mM NaCl) were recorded by measuring the absorbance at 285 nm. All cells were scanned simultaneously against the buffer containing the same additives. The time interval between scans was 3 min. The sedimentation coefficients were estimated from the differential sedimentation coefficient distribution [*c*(*s*) versus *s*] or [ls-g* (*s*) versus *s*], which were analyzed using SEDFIT program [43,44]. The *c*(*s*) analysis was performed with regularization at a confidence level of 0.68 and a floating frictional ratio. The sedimentation coefficients were corrected to the standard conditions (a solvent with the density and viscosity of water at 20°C) using SEDFIT and SEDNTERP programs [45]. Weight-average sedimentation coefficients (*s*_av_) were obtained by integration of the *c*(*s*) distribution. Molecular mass of intact BSA was calculated from *c*(*M*) distribution using SEDFIT program.

### Data Analysis

OriginPro 8.0 SR0 software (OriginLab Corporation, USA) was used for the calculations. To characterize the degree of agreement between the experimental data and calculated values, we used the coefficient of determination *R*^2^ [46].

## Results and Discussion

### Kinetics of Thermal Denaturation of BSA

**Fig 1:**
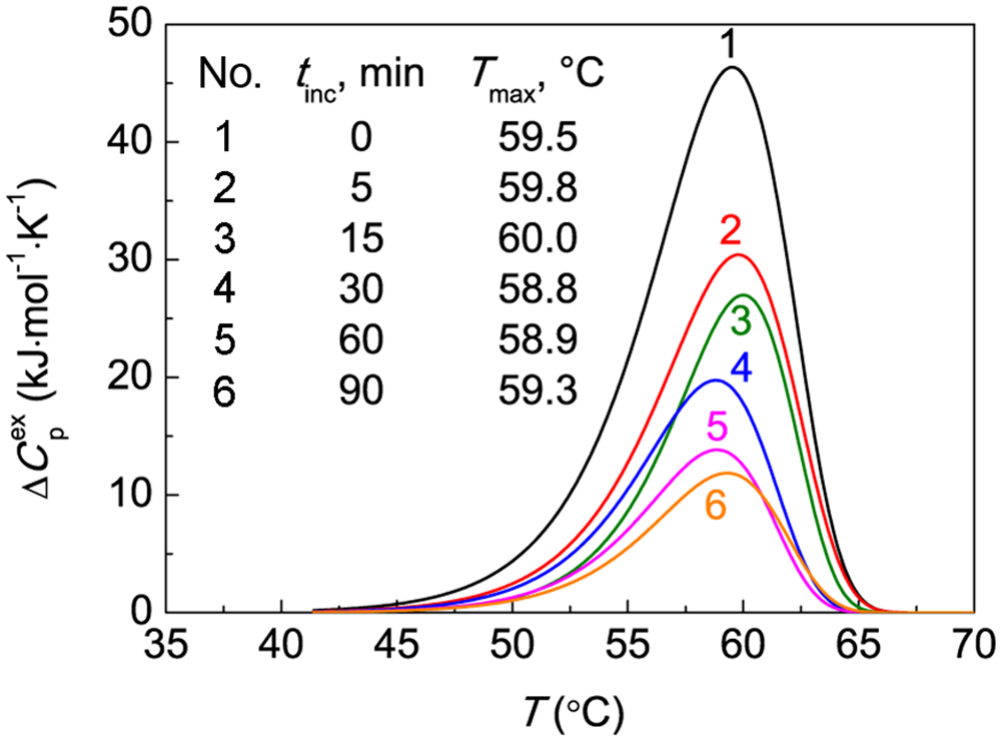
DSC profiles for BSA (1 mg/ml) preheated at 60°C (0.1 M Na-phosphate buffer, pH 7.0). The dependences of the excess heat capacity (Δ$C_{p}^{{}^{\text{ex}}}$) on temperature for BSA preincubated at 60°C for different time intervals (*t*_inc_): (1) 0, (2) 5, (3) 15, (4) 30, (5) 60 and (6) 90 min. Each DSC profile is the average of three measurements. The heating rate was 1°C/min. The inserted table gives the value of *T*_max_ for preheated BSA preparations.

DSC was used to monitor the kinetics of thermal denaturation of BSA in 0.1 M Na-phosphate buffer, pH 7.0. Fig 1 shows the results of the study of BSA stability by DSC. Curve 1 in this figure corresponds to DSC profile for original preparation of BSA. The position of the maximum (*T*_max_) on the dependence of the excess heat capacity on temperature was found to be 59.5±0.1°C. BSA solution was heated at 60°C for different intervals of time (0–90 min). The preheated solutions were cooled to room temperature and the amount of the remaining native protein was determined using DSC. Curves 2–6 in Fig 1 correspond to DSC profiles for preheated BSA samples. *T*_max_ values for these DSC profiles fall in the interval from 58.8°C to 60.0°C (see inserted table in Fig 1) and consequently are close to *T*_max_ value for the untreated BSA sample. It is assumed that denaturation heat (*Q*) expressed by the area under DSC profile is proportional to the amount of the native protein. In this case *Q*/*Q*_0_ ratio (*Q*_0_ is the denaturation heat for the original preparation of BSA) gives the portion of the protein remaining in the native state (γ_nat_) during preheating.

**Fig 2:**
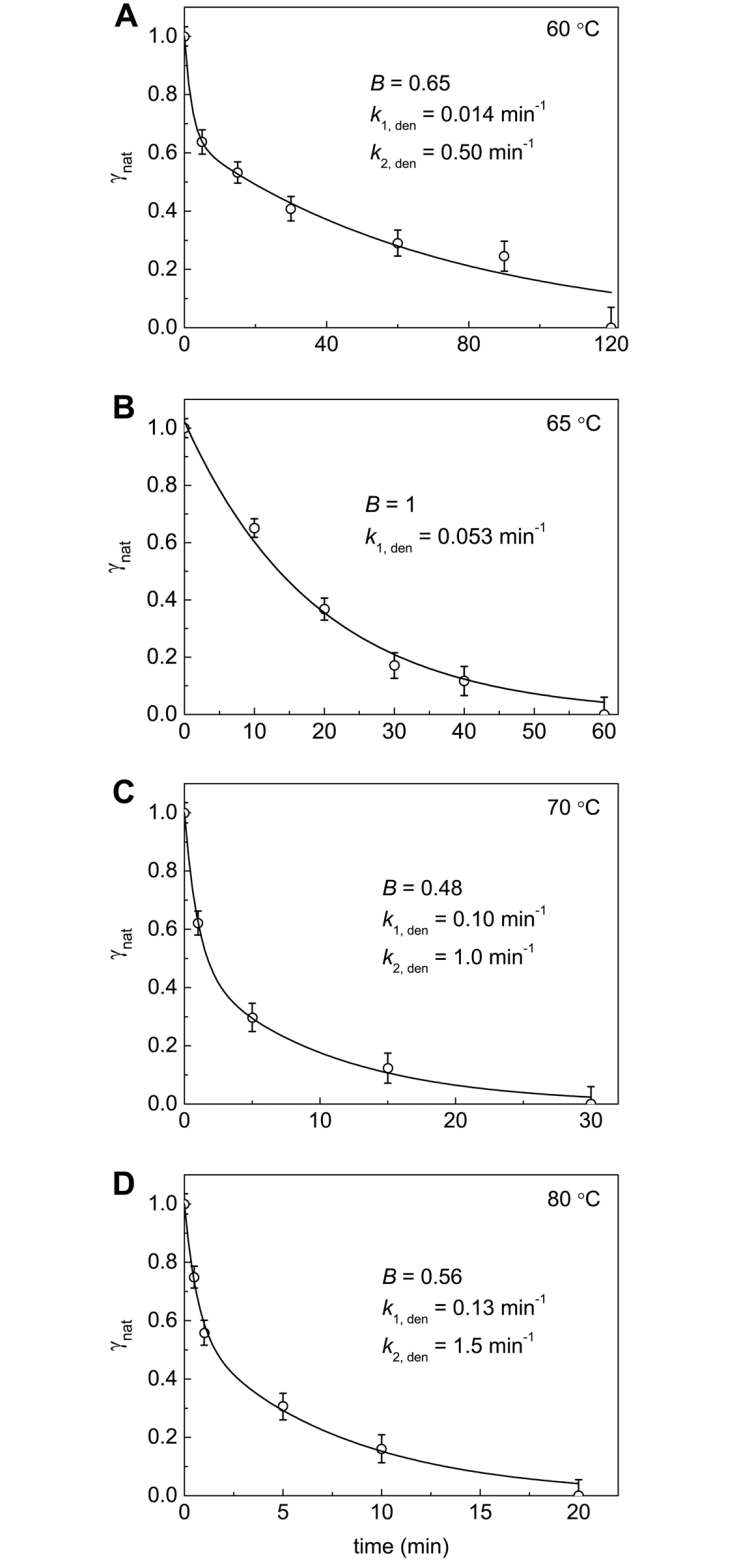
Kinetics of thermal denaturation of BSA at (A) 60°C, (B) 65°C, (C) 70°C and (D) 80°C. The dependences of the portions of the native protein (γ_nat_) on time. The γ_nat_ value was calculated as a *Q*/*Q*_0_ ratio (*Q*_0_ and *Q* are the denaturation heats determined from the area under the DSC profiles). The solid curves were calculated from the Eq 4 containing parameters *B*, *k*_1,den_ and *k*_2,den_.

The dependences of γ_nat_ on time demonstrating the kinetics of irreversible denaturation of BSA at 60°C, 65°C, 70°C and 80°C are represented in Fig 2. These dependences were approximated by the empiric equation containing two exponents:

$$\gamma_{\text{nat}}\text{ = }B\cdot \text{exp}(-k_{1\text{,den}}t)\;+\;(1\;-\;B)\cdot \text{exp}(-k_{2\text{,den}}t),$$ (4)

where *B* is the amplitude of the exponential term corresponding to the rate constant *k*_1,den_. The values of parameter *B* and rate constants *k*_1,den_ and *k*_2,den_ calculated from Eq 4 are given near the curves in Fig 2A–2D. Eq 4 can be used for calculation of the interpolated values of γ_nat_ at different times.

In order to compare thermostability of BSA at different temperatures, parameter $t_{0.5}^{\text{den}}$ (the time of half-conversion) can be used. When the temperature increases from 60°C to 80°C, the 13-fold decrease in the $t_{0.5}^{\text{den}}$ value was observed (Table 1).

**Table 1:** Parameters of thermal denaturation and aggregation of bovine serum albumin ([BSA] = 1 mg/ml, 0.1 M Na-phosphate buffer, pH 7.0).

*T*, °C	$t_{0.5}^{\text{den}}$, min	γ_non-agg,lim_	γ_Uhr_	γ_Ulr,agg_	*R*_h,1_, nm	*R*_h,2_, nm	*I*_2_, counts/s
**60**	19±1	0.51±0.01	γ_Uhr_ + γ_Ulr,agg_ = 0.49±0.01		–	11.1±0.1	(3.2±0.1)∙10^3^
**65**	13.6±0.6	0.35±0.01	0.51±0.03	0.14±0.03	10.3±0.3	12.8±0.3	(5.1±0.1)∙10^3^
**70**	1.6±0.1	0.05±0.01	γ_Uhr_ + γ_Ulr,agg_ = 0.95±0.01		10.4±0.4	15.1±0.4	(12.1±0.1)∙10^3^
**80**	1.5±0.1	0.06±0.01	γ_Uhr_ + γ_Ulr,agg_ = 0.94±0.01		–	–	–

Designations: $t_{0.5}^{\text{den}}$ is the time of half-conversion for the kinetic curves of BSA denaturation, γ_non-agg,lim_ is the portion of the unfolded protein that remains unincorporated in the large-sized aggregates for a long time (this species corresponds to A_st_ in Fig 6), γ_Uhr_ is the portion of the highly reactive unfolded form, γ_Ulr,agg_ is the portion of the low reactive unfolded form that is involved in the aggregation process by the attachment to the primary aggregates, *I*_2_ is the value of the light scattering intensity reached after completion of the formation of the secondary aggregates, *R*_h,1_ and *R*_h,2_ are the hydrodynamic radius of the primary and the secondary aggregates, respectively.

### Study of the Kinetics of Thermal Aggregation of BSA Using AF4

**Fig 3:**
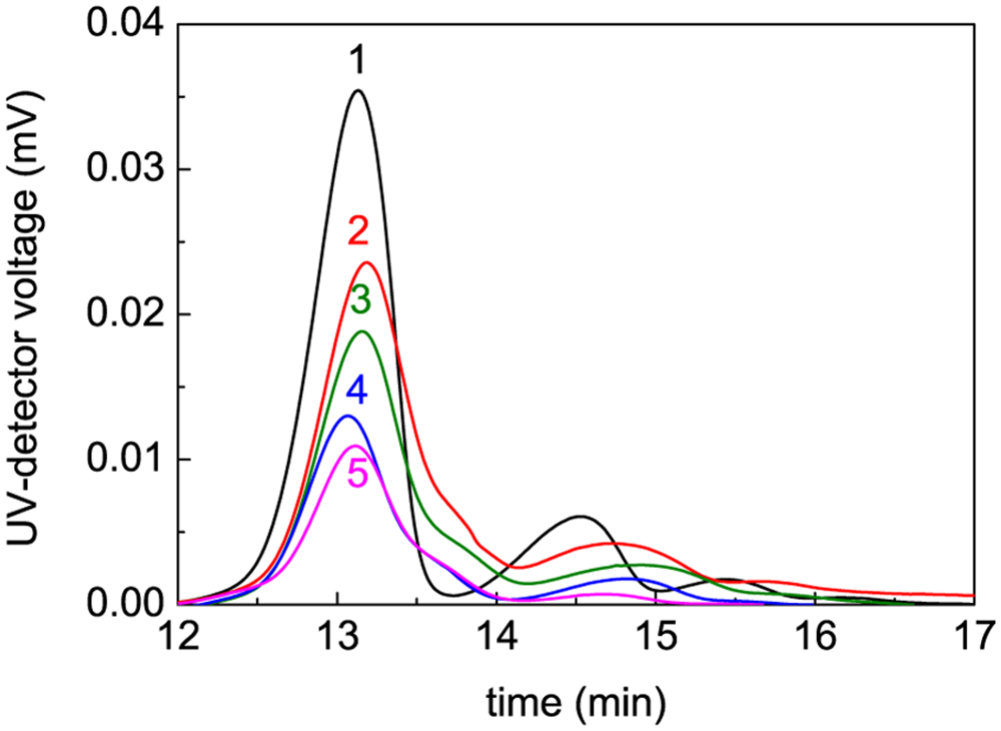
Fractograms of BSA (1 mg/ml) preheated at 65°C. The heating times were the following: (1) 0, (2) 5, (3) 15, (4) 90 and (5) 600 min. AF4 conditions: 23°C, axial (detector) flow 1 ml/min, focus flow 5 ml/min, cross flow 5 ml/min for 10 min and then linear decay to 0.1 ml/min within 20 min plus 8 min at 0 ml/min.

When studying the kinetics of thermal aggregation of BSA, portion of the non-aggregated protein was determined using AF4. As an example, Fig 3 shows fractograms of intact BSA (curve 1) and BSA preheated at 65°C for different time intervals (curves 2–5). The main peak for intact BSA corresponds to the monomeric form. There are two additional peaks corresponding to dimeric and trimeric forms. The total area under all the peaks (in the interval of the elution time from 12 to 17 min) gives the amount of the intact BSA. It should be noted that this interval of the elution times corresponds to the molecular masses of the particles from 64.5 to about 200 kDa [47]. In the case of preheated BSA the protein quantified from an area under fractogram in the above-mentioned interval of the elution time was arbitrarily called “non-aggregated protein”. It is evident that we are dealing with the protein remaining unincorporated in the large-sized aggregates. The characteristics of the non-aggregated protein will be given in the following sections.

**Fig 4:**
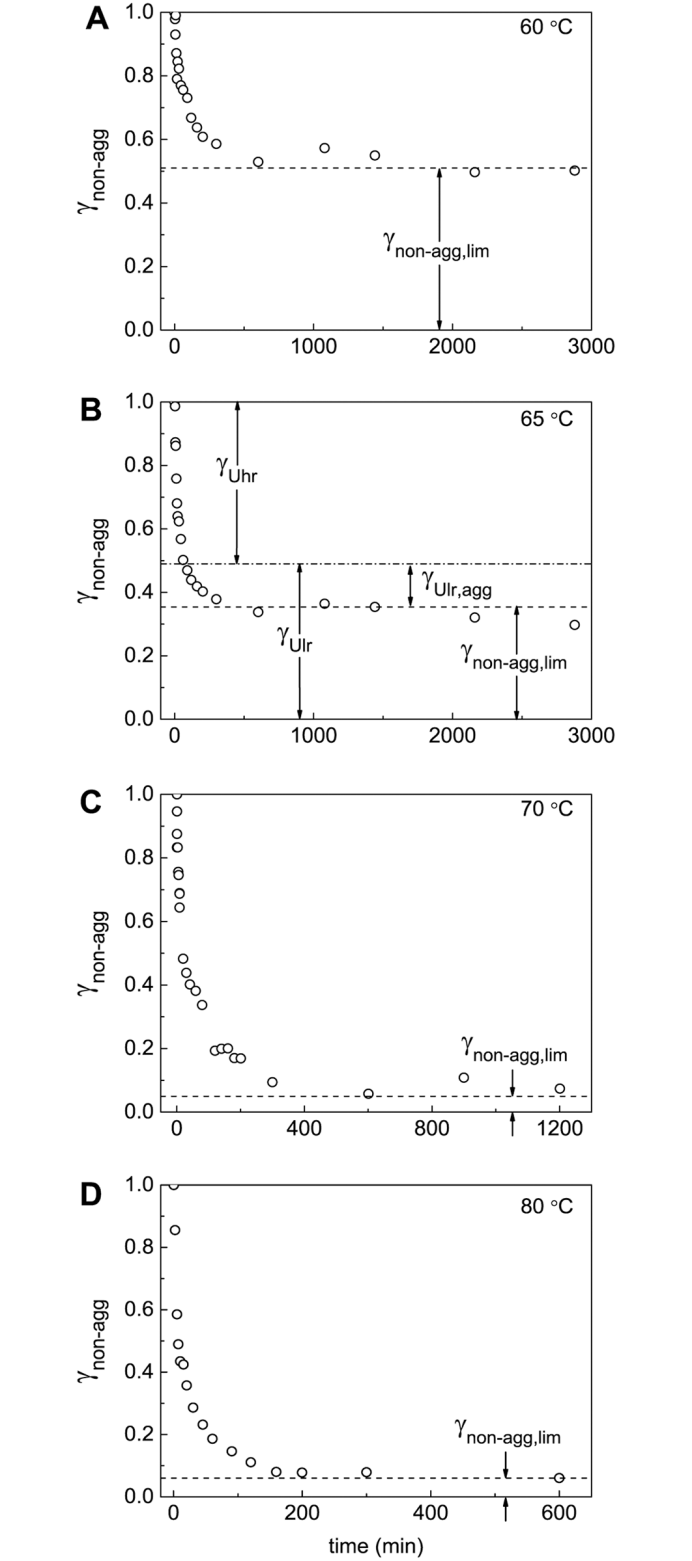
Kinetics of thermal aggregation of BSA at (A) 60°C, (B) 65°C, (C) 70°C and (D) 80°C. The dependences of portions of the non-aggregated protein (γ_non-agg_) on time. The γ_non-agg_ values were calculated from the AF4 data. The dotted horizontal lines correspond to the γ_non-agg,lim_ values. The dash-dotted line in panel B corresponds to the value of γ_Ulr_ = 1 –γ_Uhr_.

The dependences of the portion of the non-aggregated protein (γ_non-agg_) on time obtained at 60°C, 65°C, 70°C and 80°C are represented in Fig 4. It is of special interest that at rather high values of time the portion of the non-aggregated protein at each temperature approaches the limiting value different from zero (γ_non-agg,lim_). These limiting values designated as the dotted lines in Fig 4A–4D were determined by the extrapolation of the γ_non-agg_ values to *t* → ∞ in the coordinates {γ_non-agg_; 1/*t*}. The γ_non-agg,lim_ values calculated in this way correspond to the portion of the unfolded protein that remains unincorporated in the large-sized aggregates for a long time. When the temperature of incubation increases from 60°C to 80°C, the γ_non-agg,lim_ value decreases from 0.51 to 0.06 (Table 1).

**Fig 5:**
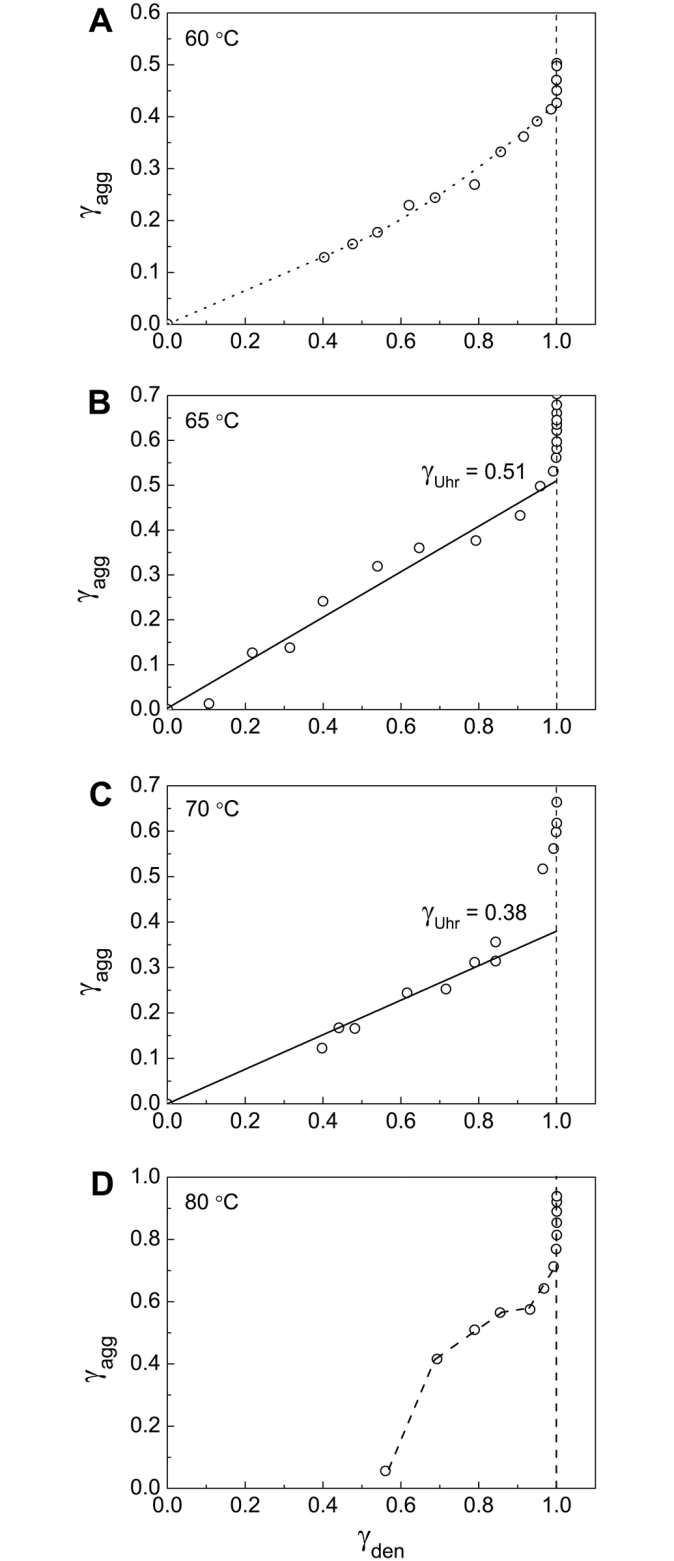
Relationship between portions of the aggregated protein (γ_agg_) and the denatured protein (γ_den_). The γ_agg_ *vs* γ_den_ plots are constructed at (A) 60°C, (B) 65°C, (C) 70°C and (D) 80°C. The values of γ_den_ = 1 –γ_nat_ were calculated from Eq 4. For each temperature parameters *B*, *k*_1,den_ and *k*_2,den_ indicated in corresponding panels of Fig 2 were used. The γ_agg_ *vs* γ_den_ plot at 65°C (panel B) was used for determination of the portion of the highly reactive BSA form (γ_Uhr_) at this temperature.

Valuable information on the initial stage of BSA aggregation can be obtained from the portion of the aggregated protein versus the portion of the denatured protein plots. The portion of the aggregated protein (γ_agg_) was calculated as (1 –γ_non-agg_) and the portion of the denatured protein (γ_den_) was calculated as (1 –γ_nat_). Fig 5 shows the γ_agg_ *vs* γ_den_ plots obtained at 60°C, 65°C, 70°C and 80°C. Particular attention has been given to Fig 5B (65°C). At this temperature the relationship between γ_agg_ and γ_den_ is linear. This means that unfolded protein rapidly aggregates without accumulation in the solution (the rate of aggregation significantly exceeds the rate of denaturation). The length cut off on the vertical line passing through γ_den_ = 1 by the linear dependence of γ_agg_ on γ_den_ gives the portion of the unfolded protein participating in fast aggregation stage (γ_Uhr_, the portion of the highly reactive unfolded protein). At 65°C γ_Uhr_ is equal to 0.51±0.03 (Table 1). The remainder of the protein (1 –γ_Uhr_) is involved in the aggregation process with the relatively low rate and can be called the low reactive unfolded protein (γ_Ulr_). At 65°C γ_Ulr_ is equal to 0.49. Taking into account that at this temperature γ_non-agg,lim_ = 0.35, we can conclude that the portion of the low reactive unfolded form involved in the formation of large-sized aggregates (γ_Ulr,agg_) is equal to 1 –γ_Uhr_−γ_non-agg,lim_ = 0.14 (Table 1).

**Fig 6:**
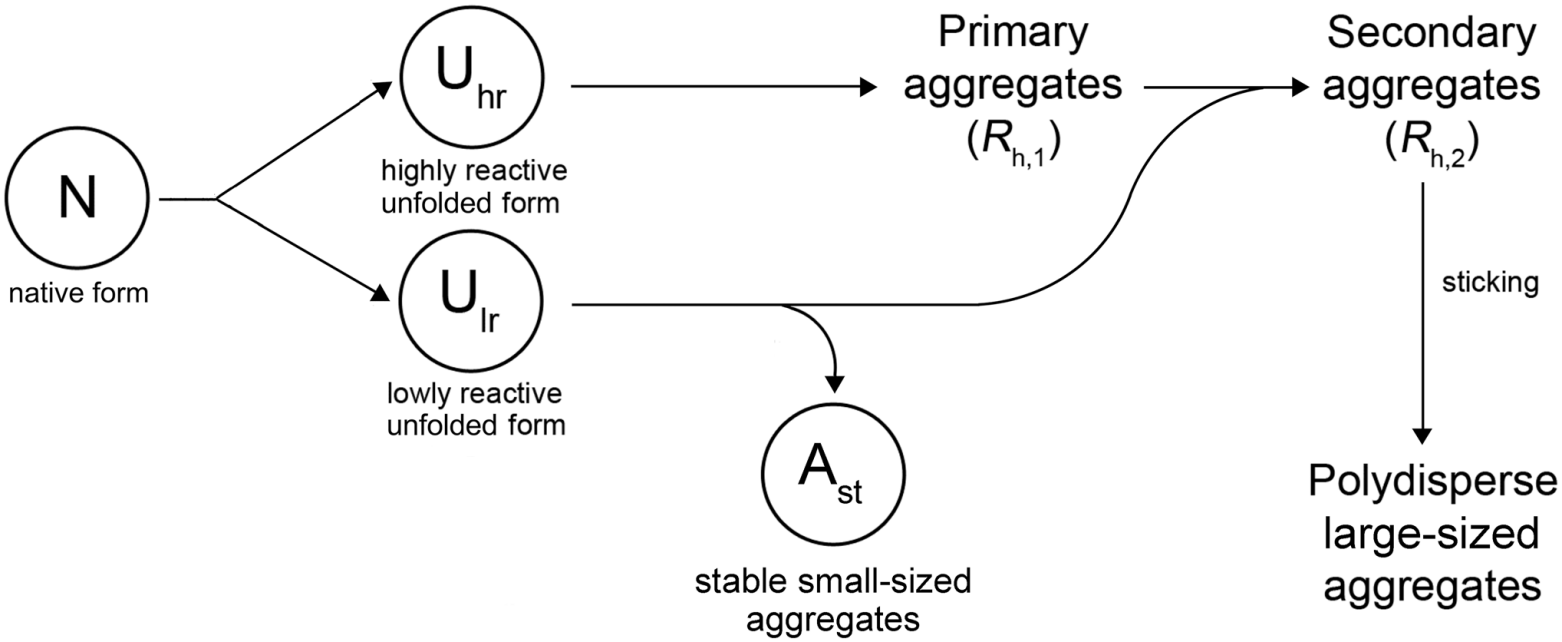
Mechanism of thermal aggregation of BSA. The first step of a general aggregation process is unfolding of the native form (N), which results in the formation of two forms of the unfolded protein with different propensity to aggregation. One of the forms (highly reactive unfolded form, U_hr_) is characterized by a high rate of aggregation; aggregation leads to formation of primary aggregates with the hydrodynamic radius (*R*_h,1_). The second form (low reactive unfolded form, U_lr_) participates in the aggregation process by its attachment to the primary aggregates produced by the U_hr_ form and possesses ability for self-aggregation with formation of stable small-sized aggregates (A_st_). The A_st_ form corresponds to non-aggregated unfolded species of BSA in AF4 experiments. At full exhaustion of the U_lr_ form, secondary aggregates with the hydrodynamic radius (*R*_h,2_) are formed. Further aggregation of the protein is a result of sticking of the secondary aggregates.

On the basis of analysis of the relationship between the portion of an aggregated protein form and the portion of a denatured form one can assume that denaturation of the native form (N) results in the formation of two unfolded species revealing different propensity to aggregation (Fig 6).The highly reactive form (U_hr_) is characterized by a high rate of aggregation. The low reactive unfolded form (U_lr_) is involved in the aggregation process through the attachment to the large-sized aggregates or formation of stable small-sized aggregates as a result of self-aggregation. In a recent paper by Rombouts et al. [48] it was shown that heating of BSA resulted in reshuffling of disulfide bonds in the protein molecule through SH—SS interchange reactions. It is highly probable that U_hr_ and U_lr_ forms differ in disulfide pairing.

### The Study of the Kinetics of Thermal Aggregation of BSA Using DLS

**Fig 7:**
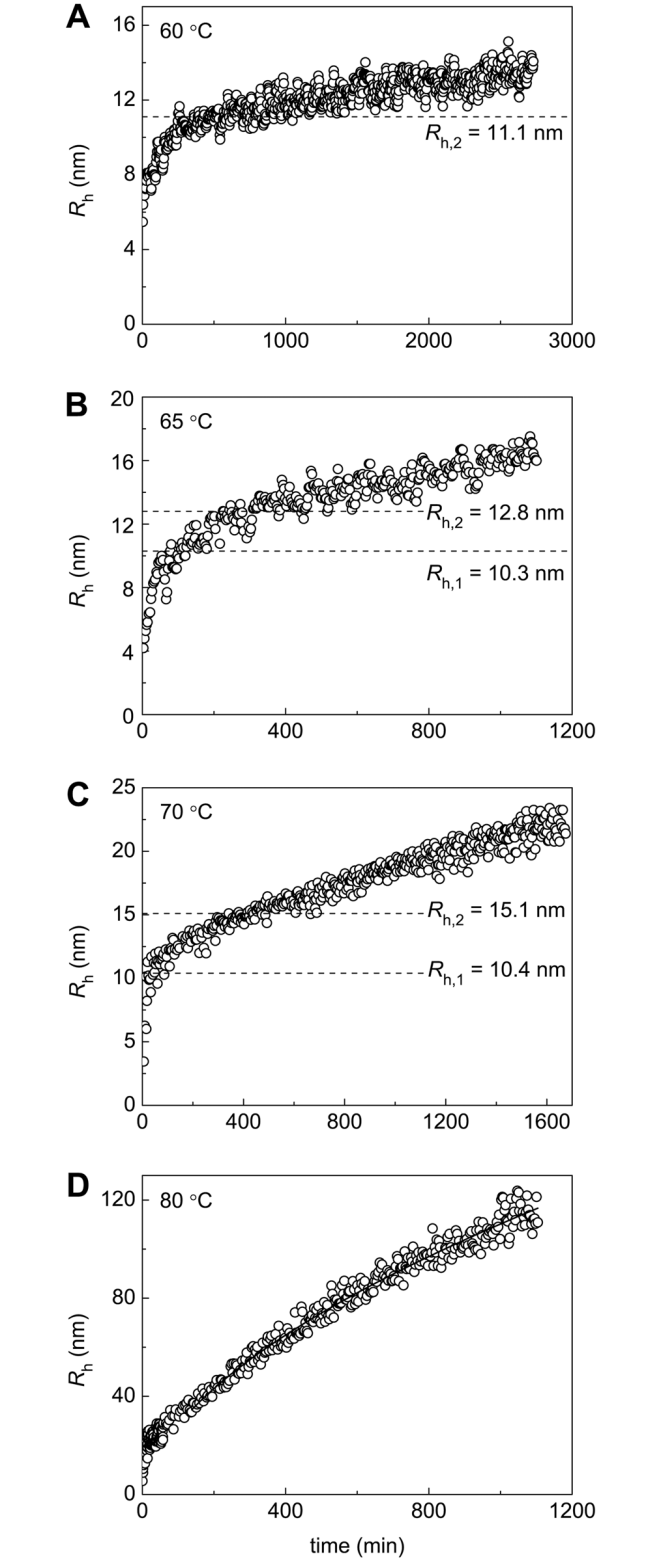
Dependences of the hydrodynamic radius (*R*_h_) on time for aggregation of BSA at (A) 60°C, (B) 65°C, (C) 70°C and (D) 80°C. The dotted horizontal lines on panels A, B, and C correspond to *R*_h,1_ or *R*_h,2_ values calculated from the dependences of *R*_h_ on the portions of denatured and aggregated BSA, respectively. The solid curve on panel D was calculated from Eq 3 at *d*_f_ = 1.76.

**Fig 8:**
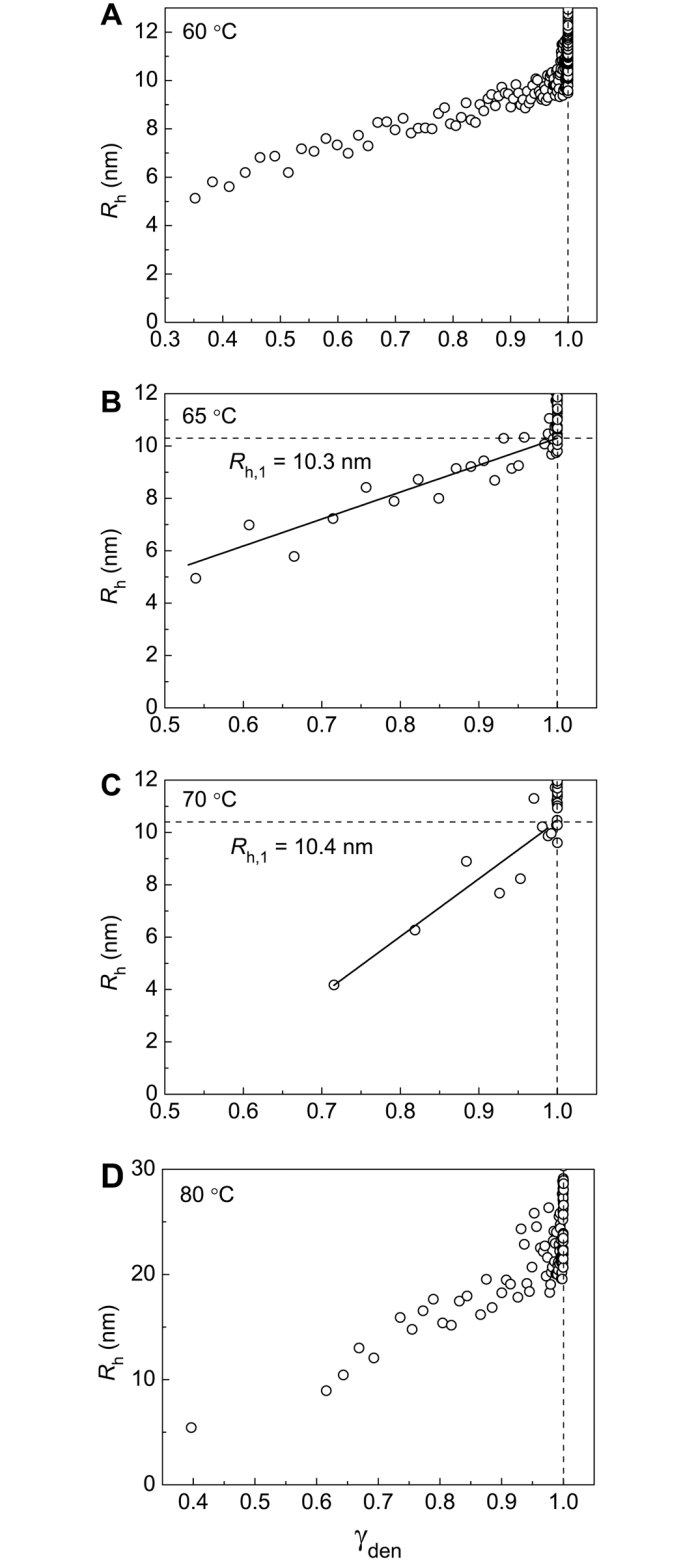
Dependences of the hydrodynamic radius (*R*_h_) on the portion of the denatured protein (γ_den_) for aggregation of BSA at (A) 60°C, (B) 65°C, (C) 70°C and (D) 80°C. The values of γ_den_ were calculated from Eq 4. For each temperature parameters *B*, *k*_1,den_ and *k*_2,den_ indicated in corresponding panels of Fig 2 were used. The *R*_h_ *vs* γ_den_ plots at 65°C and 70°C (panels B and C) were used for determination of the hydrodynamic radius of the primary aggregates (*R*_h,1_).

Additional information on the kinetics of BSA aggregation was obtained by DLS. This method allows registering changes in the light scattering intensity and hydrodynamic radius (*R*_h_) of protein aggregates in the course of aggregation. It is expedient first to discuss the dependences of *R*_h_ on time. These dependences are represented in Fig 7. As mentioned above, the highly reactive unfolded form is converted into aggregated state when denaturation is completed. The formed aggregates can be called primary aggregates (Fig 6). To estimate the hydrodynamic radius of the primary aggregates (*R*_h,1_), *R*_h_ versus portion of the denatured protein (γ_den_) plots should be constructed. This procedure is valid at temperatures of 65°C and 70°C (Fig 8B and 8C). The length cut off on the vertical line passing through γ_den_ = 1.0 by the linear dependence of *R*_h_ on γ_den_ corresponds to *R*_h,1_ value: *R*_h,1_ = 10.3±0.3 nm at 65°C and *R*_h,1_ = 10.4±0.4 nm at 70°C. It should be noted that at 60°C and 80°C (Fig 8A and 8D) determination of the size of the primary aggregates is unfeasible.

As it was pointed out above, at 65°C a part of the low reactive unfolded form (designated as γ_Ulr,agg_) can be involved in the aggregation process by the attachment to large-sized aggregates. According to Fig 6, we assume that U_lr_ is attached to the primary aggregates and this process is completed by formation of the secondary aggregates as U_lr_ is exhausted. The formation of the secondary aggregates can be considered as a heterogeneous nucleation [49,50]. In this case nucleus is formed on the surface of the primary aggregate, and growth of aggregate proceeds as a result of attachment of unfolded monomers to heterogeneous nucleus. Heterogeneous nucleation can explain the fact that the stages of formation of the primary and secondary aggregates are separated in time.

Thus, the primary aggregates act as seeds for further growth of aggregates by adding unfolded monomers. Such a seeding function of formed BSA aggregates was discussed recently by Sahin et al. [33].

**Fig 9:**
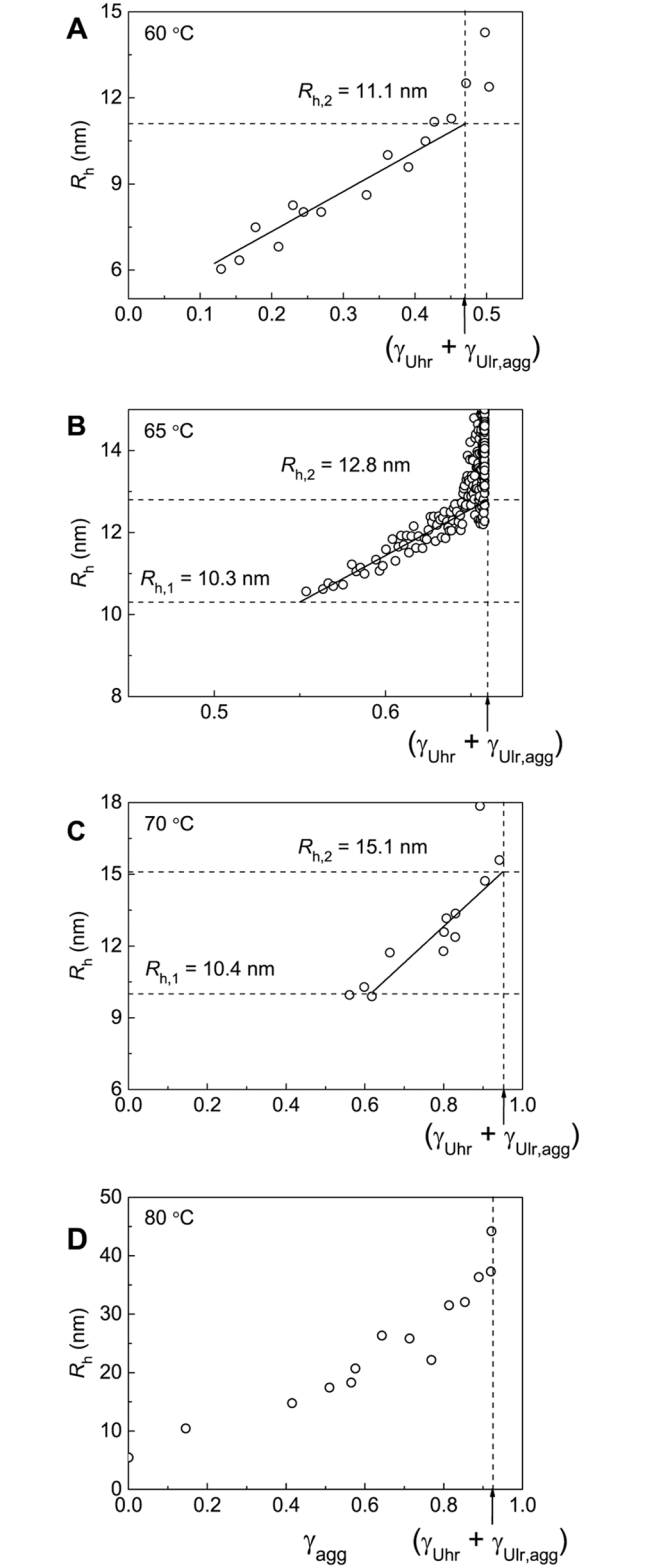
Dependences of the hydrodynamic radius (*R*_h_) on the portion of the aggregated protein (γ_agg_) for aggregation of BSA at (A) 60°C, (B) 65°C, (C) 70°C and (D) 80°C. The vertical dotted lines correspond to γ_agg_ = γ_Uhr_ + γ_Ulr,agg_. The *R*_h_ *vs* γ_den_ plots at 60°C, 65°C and 70°C (panels A–C) were used for determination of the hydrodynamic radius of the secondary aggregates (*R*_h,2_).

To calculate the hydrodynamic radius of the secondary aggregates (*R*_h,2_), *R*_h_ versus γ_agg_ plot should be constructed. The (γ_Uhr_ + γ_Ulr,agg_) value on the abscissa axis corresponds to the completion of the stage of formation of the secondary aggregates. Thus, the length cut off on the vertical line passing through γ_agg_ = (γ_Uhr_ + γ_Ulr,agg_) by the linear dependence of *R*_h_ on γ_agg_ gives the *R*_h,2_ value (11.1±0.1 nm at 60°C, 12.8±0.3 nm at 65°C and 15.1±0.4 nm at 70°C; Fig 9A–9C, Table 1).

The obtained experimental data suggest that at 65°C the aggregation process involves three stages, which are markedly separated in time, namely, BSA unfolding, formation of the primary aggregates and formation of the secondary aggregates. This circumstance allows determining the portion of the individual forms U_hr_ and U_lr_ and the size of the primary and secondary aggregates (*R*_h,1_ and *R*_h,2_) at this temperature. At 60°C the rates of denaturation and aggregation become commensurate and determination of the individual portions of highly and low reactive forms of the unfolded protein is impossible. At 70°C the rates of formation of the primary and secondary aggregates become commensurate. At 80°C analysis of the kinetics of BSA aggregation is complicated by the formation of large-sized aggregates (this problem will be discussed below). It should be noted that experimental data permit us to estimate the portion of the unfolded protein that remains unincorporated in the large-sized aggregates for a long time (γ_non-agg,lim_) at all of the temperatures under study (Fig 4A–4D). Besides, the hydrodynamic radius of the secondary aggregates (*R*_h,2_) at 60°C and 70°C can be estimated from the *R*_h_ versus γ_agg_ plots. At 80°C the determination of the size of the secondary aggregates is unfeasible.

As it can be seen from Figs 7 and 9, formation of the secondary aggregates is not the final stage of the aggregation process, and the size of aggregates continues increasing in time monotonously. Thus, scheme represented in Fig 6 should be supplemented with the stage of sticking of the secondary aggregates. Formation of large-sized aggregates becomes especially marked at 80°C. At *t* > t* = 140 min and *R*_h_ > $R_{\text{h}}^{*}$ = 35 nm the dependence of *R*_h_ on time follows Eq 3 with *d*_f_ = 1.76±0.06 indicating that sticking of aggregates proceeds in DLCA kinetic regime.

**Fig 10:**
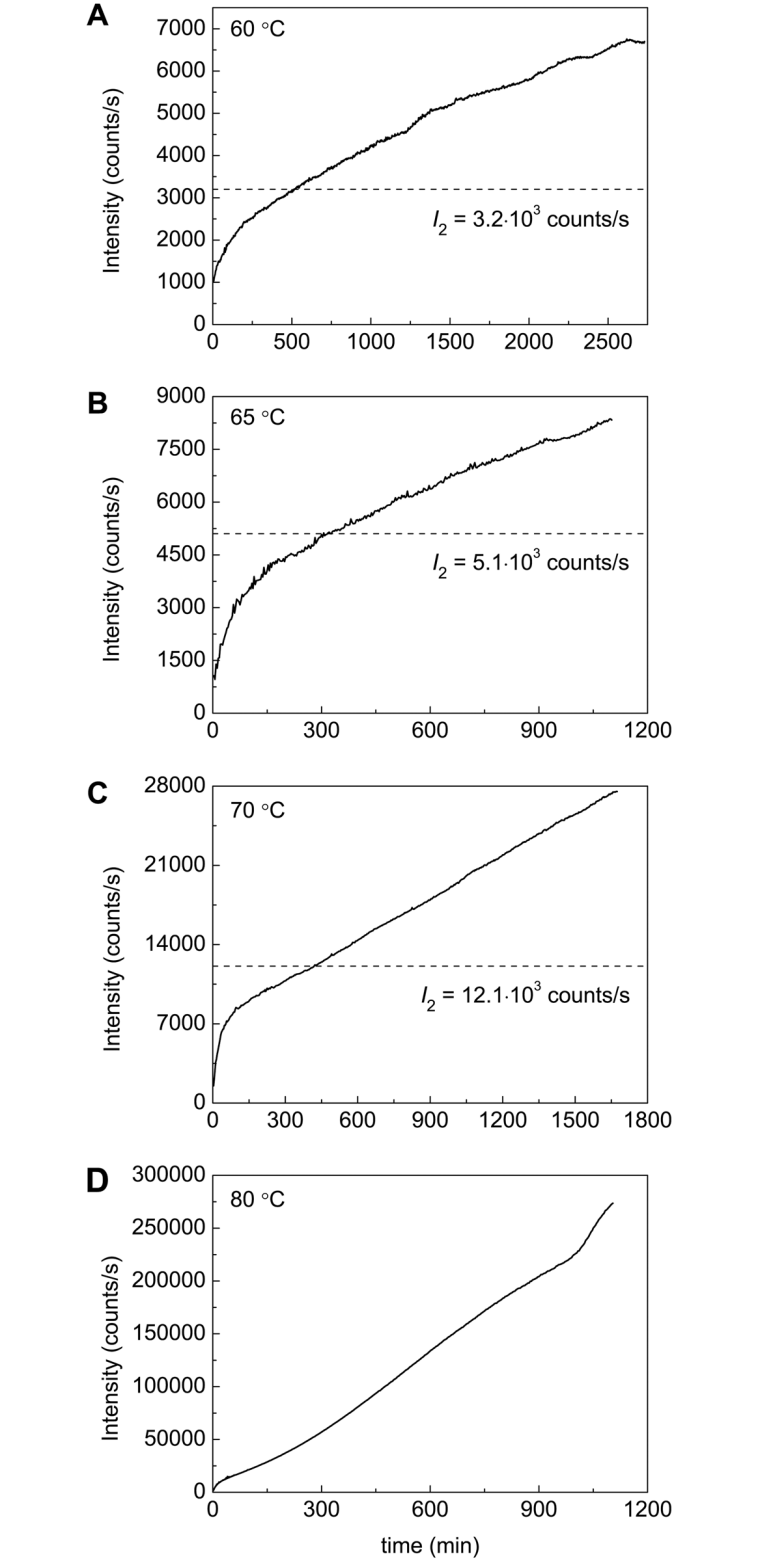
Dependences of the light scattering intensity on time for aggregation of BSA at (A) 60°C, (B) 65°C, (C) 70°C and (D) 80°C. The dotted horizontal lines on the panels A, B and C correspond to *I*_2_ values calculated from the dependences of the light scattering intensity on the portion of aggregated BSA.

**Fig 11:**
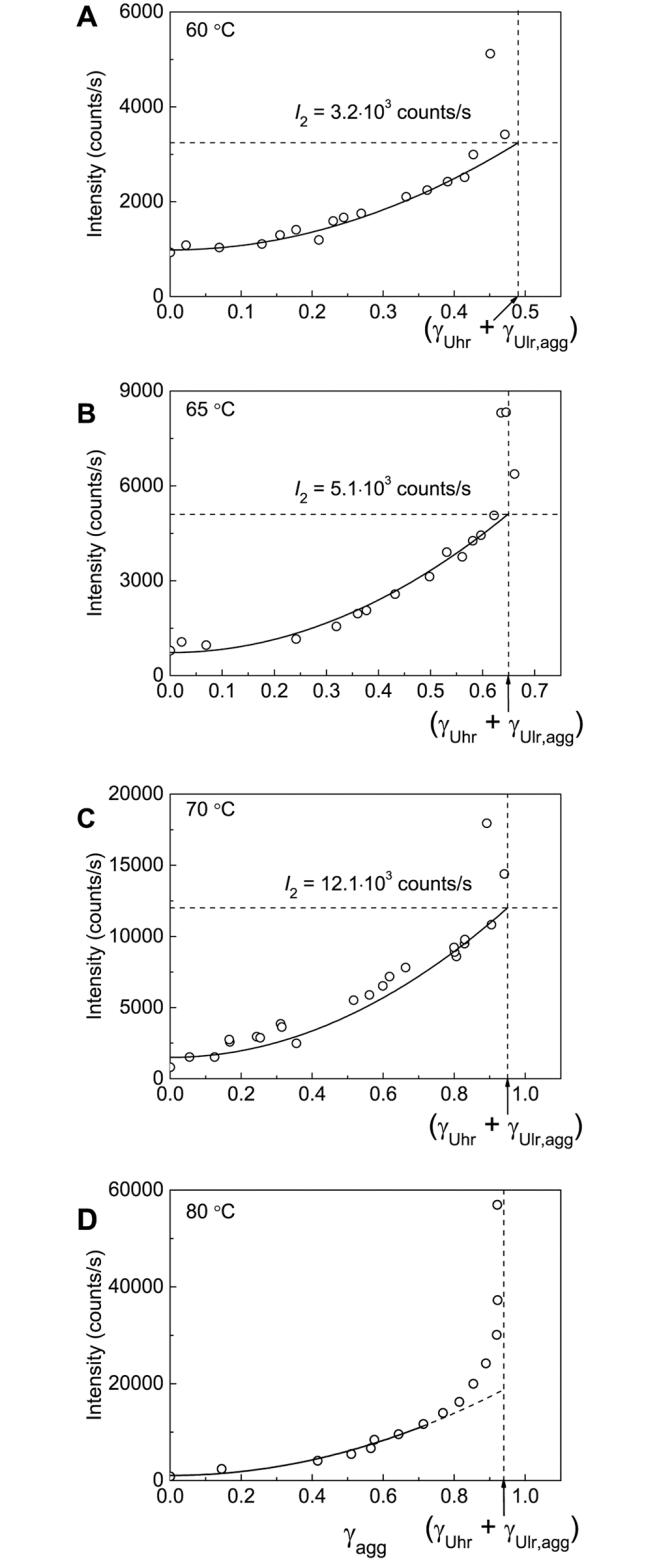
Dependences of the light scattering intensity (*I*) on the portion of the aggregated protein (γ_agg_) for aggregation of BSA at (A) 60°C, (B) 65°C, (C) 70°C and (D) 80°C. The vertical dotted lines correspond to γ_agg_ = (γ_Uhr_ + γ_Ulr,agg_). The *I vs* γ_agg_ plots at 60°C, 65°C and 70°C (panels A–C) were used for determination of parameter *I*_2_ corresponding to the value of the light scattering intensity after completion of the secondary BSA aggregates formation. The solid lines were calculated from Eq 5.

Fig 10 shows dependences of the light scattering intensity (*I*) on time obtained for BSA aggregation at 60°C, 65°C, 70°C and 80°C. As it can be seen, the *I* value increases monotonously in time. Such a monotonous increase is due to the fact that the aggregation process does not come to a stop at the stage of formation of the secondary BSA aggregates and continues as a result of sticking of the secondary aggregates. To estimate the level of the light scattering intensity (*I*_2_) corresponding to the full transition of the highly and low reactive unfolded forms into the aggregated state, the intensity versus γ_agg_ plots were constructed (Fig 11). The analysis of these plots shows that the dependence of the light scattering intensity on γ_agg_ follows the quadratic law:

$$I=I_{0}+\frac{I_{2}-I_{0}}{{\left(\gamma_{\text{Uhr}}+\gamma_{\text{Ulr,agg}}\right)}^{2}}\;\gamma_{\text{agg}}^{\text{2}},$$ (5)

where *I*_0_ is the initial value of the light scattering intensity. The following values of *I*_2_ were found: (3.2±0.1)·10^3^, (5.1±0.1)·10^3^ and (12.1±0.1)·10^3^ counts/s at 60°C, 65°C and 70°C, respectively. The dotted horizontal lines in Fig 10A–10C correspond to the calculated values of *I*_2_. It should be noted that at 80°C the reliable estimation of *I*_2_ value is impossible, although the initial part of the dependence of the light scattering intensity on γ_agg_ can be described by the quadratic law.

The fact that the light scattering intensity (*I*) and the portion of the aggregated protein (γ_agg_) are connected by quadratic equation allows us to transform the experimental dependence of *I* on time into the kinetic curve of the accumulation of the aggregated protein: γ_agg_ = const (*I*/*I*_0_−1)^0.5^. Such an approach can be used for the calculation of the initial rate of aggregation and, consequently, for screening of the agents acting as suppressors of protein aggregation.

### The Study of Polydispersity of Heated BSA Preparations Using AUC

**Fig 12:**
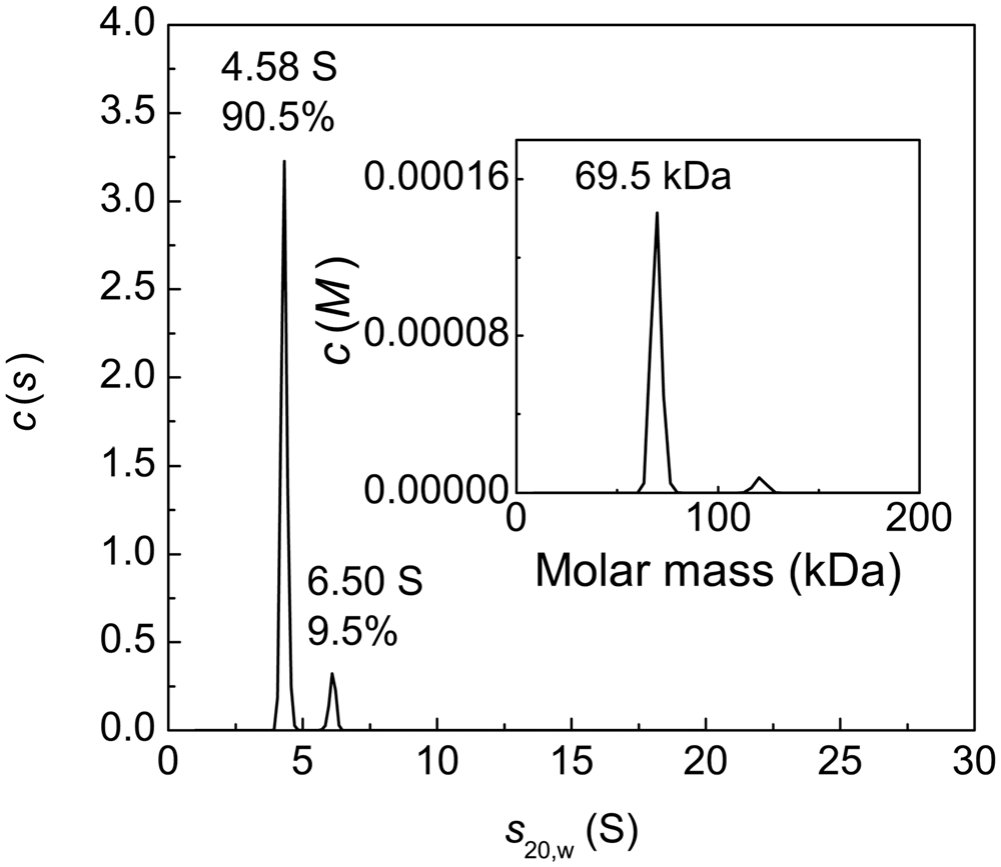
Sedimentation behavior of native BSA at 20°C. The differential sedimentation coefficient distributions *c*(*s*) for BSA (1 mg/ml). The inset shows *c*(*M*) distribution. Rotor speed was 48000 rpm.

AUC was used to characterize polydispersity of heat-treated BSA preparations. Fig 12 shows the differential sedimentation coefficients distribution *c*(*s*) for the intact preparation of BSA. The main peak with *s*_20,w_ = 4.58±0.12 S and *M* = 69.5 kDa corresponds to the monomeric form of BSA. The content of the monomeric form is 90.5%. The *c*(*s*) distribution also contains a minor peak with *s*_20,w_ = 6.50±0.11 S corresponding to the dimeric form.

**Fig 13:**
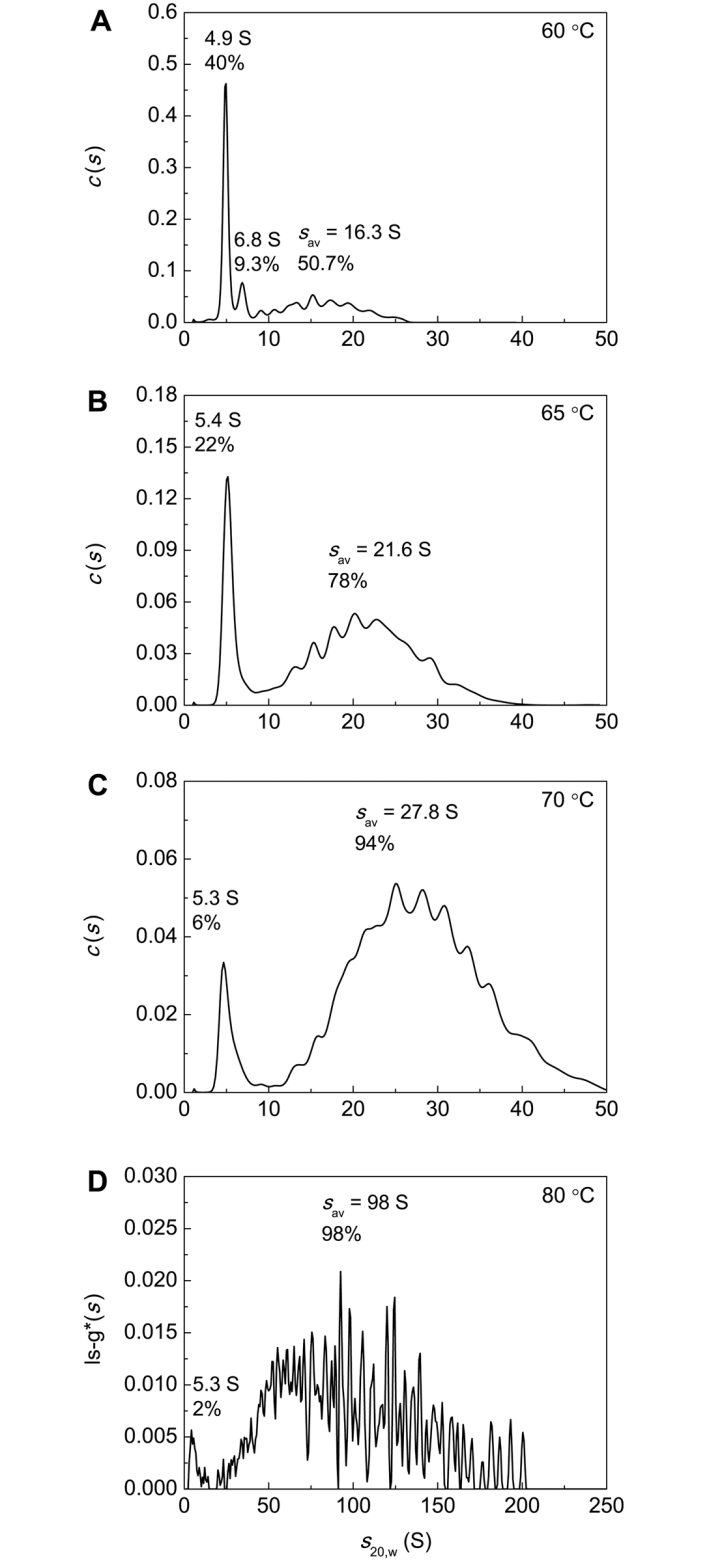
Sedimentation behavior of BSA (1 mg/ml) preincubated for 12 h at (A) 60°C, (B) 65°C, (C) 70°C and (D) 80°C. The *c*(*s*) and ls-g*(*s*) distributions were obtained at 24°C. Rotor speed was 52000 rpm.

The *c*(*s*) distributions for BSA preparation preheated for 12 h at 60°C, 65°C, 70°C and 80°C are represented in Fig 13A–13D. It should be noted that according to the data shown in Fig 4 at *t* = 12 h the portion of the non-aggregated protein reaches a constant level corresponding to the portion of the unfolded protein that remains unincorporated in the large-sized aggregates for a long time (γ_non-agg,lim_) at each value of temperature under study. In the case of BSA preparation preheated at 60°C (Fig 13A) the non-aggregated protein is represented by the monomeric form with *s*_20,w_ = 4.9±0.4 S (40%) and dimeric form with *s*_20,w_ = 6.8±0.5 S (9%). Apart from the monomeric and dimeric forms the *c*(*s*) distributions contain a set of peaks corresponding to BSA aggregates (50.7%). The latter are characterized by high polydispersity. The weight-average sedimentation coefficient (*s*_av_) for aggregates was found to be 16.3 S (Std. dev. 4.3 S). Thus, the portion of the non-aggregated protein is equal to 0.49 and agrees well with the portion of the non-aggregated protein (γ_non-agg,lim_) determined by AF4 (Fig 4A).

The *c*(*s*) distributions for BSA preheated at 65°C and 70°C (Fig 13B and 13C) and ls-g*(*s*) distribution for BSA preheated at 80°C (Fig 13D) contain peaks with *s*_20,w_ = 5.3–5.4 S corresponding to the non-aggregated protein involving unfolded monomeric and dimeric forms and broad peaks corresponding to protein aggregates. The percentage of the non-aggregated protein decreases from 21.6% to 2.4%, when the temperature of incubation increases in the interval from 65°C to 80°C.

As it can be seen from Fig 13B–13D, at higher temperatures for incubation of BSA solution higher-order aggregates are formed. The *s*_av_ values calculated for aggregated forms at 65°C, 70°C and 80°C were 21.6 S (Std. dev. 6.1 S), 27.8 S (Std. dev. 7.5 S) and 96 S (Std. dev. 38 S), respectively. The amount of aggregated forms increased concurrently with the increase in the size of protein aggregates.

### The Study of Morphology of Heat-Treated BSA Preparations Using TEM

**Fig 14:**
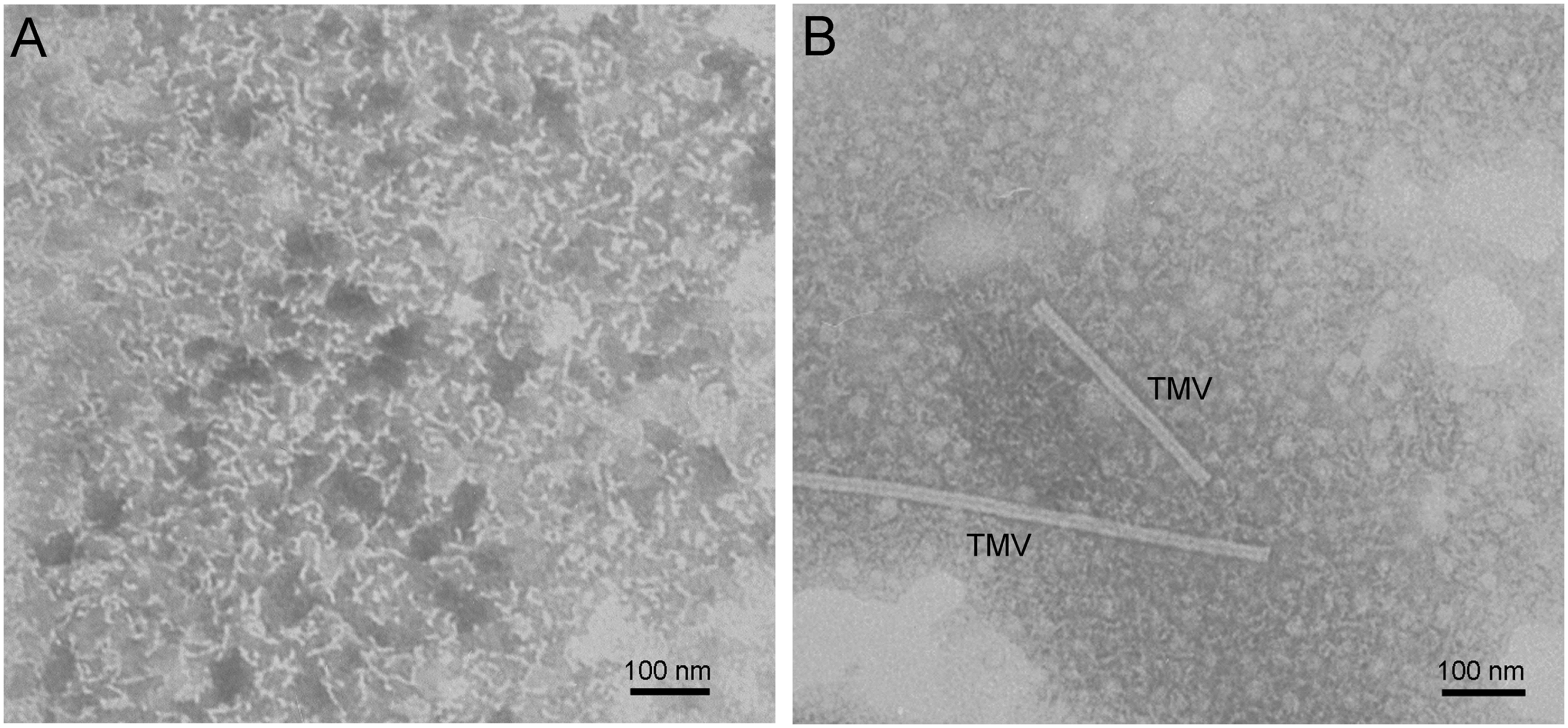
TEM images of BSA preparations pre-heated at 65°C for 110 min (A) and 330 min (B). BSA concentration was 1 mg/ml. Preparation preheated for 110 min was washed in deionized water. Image in panel B contains particles of tobacco mosaic virus (TMV).

To characterize the morphology of pre-heated BSA preparations, the structure of formed aggregates was studied using TEM. BSA preparations were incubated at 65°C for 110 or 330 min. These time intervals correspond to the completion of formation of primary or secondary aggregates, respectively (see Fig 7B). With the time of incubation equal to 110 min, the short curved fibrils with the length of about 40 nm were formed (Fig 14A). There are aggregates of different shape including fibrils, spheres and fibrils with a bulb at one end on the TEM image obtained for BSA preparation preheated for 330 min (Fig 14B). According to Fig 6, formation of these aggregates (secondary aggregates) proceeds as a result of attachment of unfolded molecules to the primary aggregates.

### Interaction of Heat-Treated BSA with ThT

**Fig 15:**
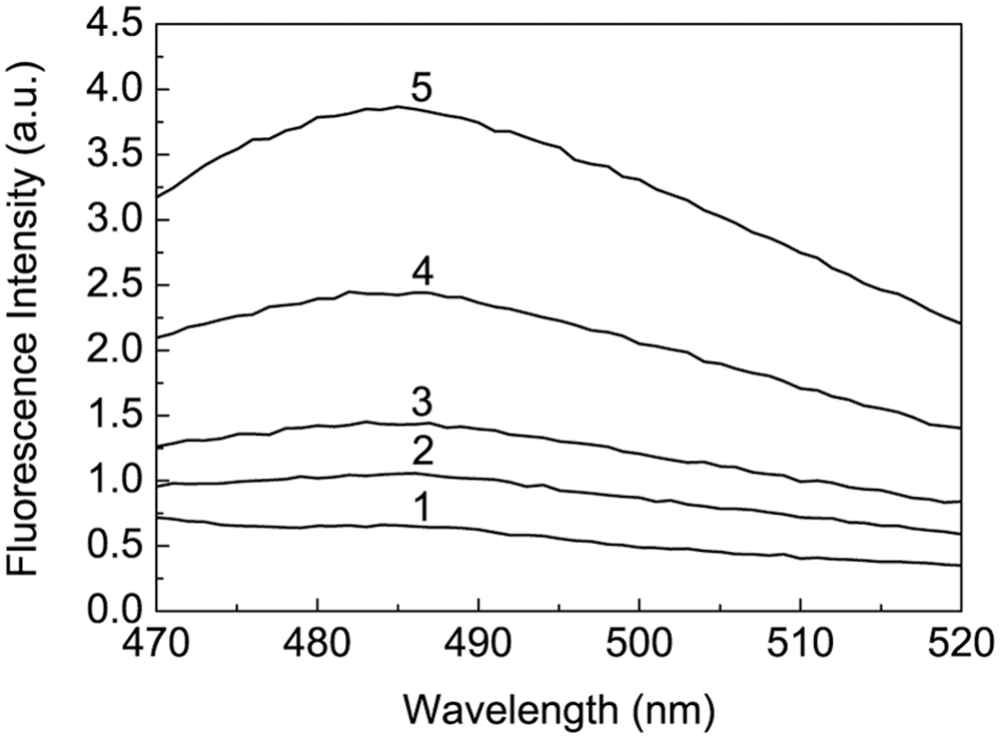
Fluorescence emission spectra of ThT (20 μM; curve 1) and ThT solution incubated with heated BSA (0.4 mg/ml) for 30 min at 25°C. BSA (1 mg/ml) was preincubated for 12 h at 60°C, 65°C, 70°C and 80°C (curves 2–5, respectively). Excitation wavelength was 450 nm.

Holm et al. [34] observed marked increase in emission of ThT fluorescence at 482 nm when fibril-specific dye was added to BSA solution incubated at 70°C (20 mM Tris, pH 7.4). Concentration of BSA in these experiments was 2.5 mg/ml. On the basis of the obtained data the authors concluded that BSA is prone to formation of amyloid-like fibrils under the studied conditions. Taking into account these results it was of interest to check amyloid fibrillation of BSA in 0.1 M Na-phosphate buffer, pH 7.0, at [BSA] = 1 mg/ml. After 12 h heating of BSA (1 mg/ml) at 60°C, 65°C, 70°C and 80°C each BSA solution was incubated with ThT for 30 min at 25°C. The final concentrations of BSA and ThT were 0.4 mg/ml and 20 μM, respectively. It is generally accepted that the significant increase in ThT fluorescence is indicative of formation of amyloid fibrils, which are characterized by cross-β-sheet rich structure [51–53]. As it can be seen from Fig 15, the insignificant changes in ThT fluorescence take place upon binding of ThT to heated BSA. The maximum increase in ThT fluorescence, namely 6-fold, was observed for BSA preincubated at 80°C. Thus, it would be unreasonable to suppose that amyloid fibrils of BSA can be formed under the conditions set for all of the experiments in the present work.

### Isolation of the Fraction of Non-Aggregated Unfolded BSA and its Characterization

To isolate the fraction of non-aggregated unfolded BSA (A_st_ in Fig 6) and to study its properties, preference was given to temperature of incubation of 60°C for BSA aggregation, where a portion of this form was maximum for the temperatures studied (γ_non-agg,lim_ = 0.51; Table 1). BSA aliquotes were added to 0.1 M Na-phosphate buffer, pH 7.0, preheated to 60°C to the final protein concentration of 1 mg/ml and incubated at 60°C for 12 h. Then the samples were cooled to room temperature (23°C) and concentrated on Microcon filter microtubes (NMWL 50000) by centrifugation at 14000 g for 10 min. The protein concentration in the supernatant was determined spectrophotometrically at 280 nm using the absorption coefficient $A_{\text{cm}}^{1\%}$ of 6.58 [39]. The obtained sample was then analyzed by SEC. The data of SEC are represented in S1 Fig. The peak with the elution time of 70.8 min (peak 1) corresponds to large-sized aggregates of BSA. The molecular mass of this fraction determined by means of the calibration plot is 300 kDa. The peak with the elution time of 86 min (peak 2) corresponds to BSA aggregates with average molecular mass of 151 kDa. The peak with the elution time of 100.7 min (peak 3) corresponds to the species with molecular mass of 77.6 kDa.

The data on AUC supports our conclusion that peak 2 is represented by small aggregates of BSA. The *c*(*s*) distribution for the fraction with elution time in the interval from 82 to 94.6 min involves the set of peaks with *s*_20,w_ ranging from 5.9 to 20.7 S (S2A Fig). The single peak with *s*_20,w_ = 5.2 S (63%) was observed for the fraction with elution time in the interval from 94.6 to 122 min (S2B Fig). One may assume that peak 3 on the SEC elution profile contains the unfolded monomeric form and small amount of the unfolded dimeric form of BSA. DLS measurements show that this fraction is characterized by the *R*_h_ value of 6.1 nm (S3B Fig). The obtained value of *R*_h_ exceeds the corresponding value for the intact BSA (3.4 nm; S3A Fig) suggesting that the higher *R*_h_ value for peak 3 is due to the presence of the dimeric form.

The tryptophan fluorescence spectrum for the SEC-obtained fraction of BSA preheated for 12 h at 60°C with elution time in the interval from 94.6 to 122 min was compared with that for intact BSA (S4 Fig). As it can be seen, the non-aggregated unfolded form (A_st_) is characterized by diminished value of emission intensity of Trp and shift of the emission maximum wavelength (λ_max_) from 346 to 337 nm. Such a blue shift is surprising because unfolding of proteins usually results in the displacement of λ_max_ towards higher wavelengths [54,55]. When heating BSA in the temperature interval from 25°C to 80°C, Sahin et al. [33] and Wen et al. [56] observed a blue shift of peak position for intrinsic tryptophan fluorescence of BSA. A blue shift of the emission spectrum for tryptophan fluorescence observed for thermal unfolding of pig pancreas α-amylase [55] and myosin subfragment 1 [57] was explained by the fact that Trp residues were introduced into a more hydrophobic environment as a result of conformational changes of the protein molecule accompanying its unfolding. One may assume that the observed blue shift of λ_max_ for A_st_ form of BSA is due to transference of Trp residues into more hydrophobic surroundings induced by dimerization of unfolded monomers.

Additional information on the changes in the accessibility of hydrophobic sites in the BSA molecule during heat treatment can be obtained using fluorescent probe. The position of λ_max_ in the emission spectrum for free ANS corresponds to 528 nm (excitation wavelength was 445 nm). To study the interaction of BSA with ANS, we used the excitation wavelength of 380 nm [56]. Under studied conditions ANS fluorescence is negligible (curve 1 in S5 Fig), however it markedly increases in the presence of intact BSA suggesting that there are numerous ANS-binding sites in the protein molecule. Binding of ANS with hydrophobic sites in the BSA molecule results in the shift in the position of λ_max_ to 484 nm (curve 2). Interestingly, the increment in ANS fluorescence intensity in the presence of non-aggregated unfolded fraction of BSA (curve 3) is markedly lower than in the case of intact BSA. The position of λ_max_ in the system A_st_ + ANS corresponds to 476 nm. Thus, formation of A_st_ species is not accompanied by multiplication of additional hydrophobic sites in the BSA molecule.

For estimation of colloidal stability of native and non-aggregated unfolded BSA, zeta potential of both preparations was measured. The values of zeta potential for untreated BSA and A_st_ were found to be –20.9±0.8 and –9.2±0.4 mV, respectively. The zeta potential for native protein at pH and ionic strength used in our experiments is consistent with literature data [58]. The lower absolute value of zeta potential for A_st_ suggests that non-aggregated unfolded form would be prone to aggregation due to weaker electrostatic repulsion. Possible explanation of the inability of A_st_ to form aggregates under our experimental conditions is that, as demonstrated by the experiments with fluorescence probe ANS, there is no emergence of additional hydrophobic sites during unfolding of native form.

The CD spectra of native and non-aggregated unfolded BSA are represented in S6 Fig. The content of the secondary structure elements was estimated using software available online on DichroWeb server (http://dichroweb.cryst.bbk.ac.uk). For calculation we used two analysis programs, CONTIN [59] and SELCON [60], with two protein references sets (set 3 and SP175, 190–240). The results obtained with different sets of reference proteins were similar. The averages of all matching solutions for both programs are given in S1 Table. The normalized root mean square deviation (NRMSD) values for both fittings were found not exceeding 0.1 [40].

The data obtained by CONTIN and SELCON program show that the portion of α-helices in the secondary structure of untreated BSA is equal to 0.49. BSA in its native state predominantly has α-helical structure [9], and that is consistent with our results. Preheating at 60°C for 12 h leads to a noticeable decrease in the portion of α-helices by 22–37% and an increase in the portion of β-strands by 27–60%, the portion of turns by 13–25% and the portion of unordered structural elements by 12–24%.

The observed changes in the content of α-helices are typical of heat-treated albumin. Literature data show that heating of BSA to 65°C at a constant rate [61] or incubation at 90°C for 10 min [62] are accompanied by a decrease in the portion of α-helices. Zhang et al. [63] showed that the secondary structure of BSA could be recovered after heating to 79.42°C and subsequent cooling to the room temperature. It should be noted that thermal denaturation of BSA involves reversible and irreversible stages [64]. Under experimental conditions used in the present work (heating for 12 h at 60°C) BSA was denatured completely and irreversibly, as exemplified by the data represented in Fig 2A that shows an irreversible stage of denaturation.

## Conclusion

Analysis of the relationship between the portions of aggregated and denatured protein allowed us to make a conclusion about the kinetic regime of thermal aggregation of proteins. Using this approach, we have demonstrated that the rate-limiting stage of BSA aggregation at 65°C is the stage of protein unfolding. At higher temperatures (for example, at 80°C) the stage of aggregation of the denatured protein molecules becomes the rate-limiting stage.

Earlier we have shown that a test system based on dithiothreitol-induced aggregation of BSA can be used for testing the anti-aggregation activity of chaperones of proteinous nature and low-molecular-weight chemical chaperones [47]. The results obtained in the present paper demonstrate that heat-induced aggregation of BSA can also be useful for the development of a test system designed for screening the agents, which reveal an ability to suppress aggregation of proteins. The basis for this intention is the fact that for the initial parts of the kinetic curves of thermal aggregation of BSA the light scattering intensity is proportional to the portion of the aggregated protein raised to the second power. Thus, measurements of the time-dependent changes in the light scattering intensity allow the quantitative estimation of the anti-aggregation activity of agents under study to be carried out.

As for the mechanism of thermal aggregation of BSA, it is notable that the character of dependence of the hydrodynamic radius of protein aggregates on time for BSA aggregation differs essentially from that for thermal aggregation of other proteins studied by us earlier. When studying thermal aggregation of GAPDH, glycogen phosphorylase *b* and creatine kinase from rabbit skeletal muscles, β_L_-crystallin, tobacco mosaic virus coat protein, yeast alcohol dehydrogenase and mitochondrial aspartate aminotransferase using DLS, we have shown that the initial stage of protein aggregation is that of formation of start aggregates involving hundreds of denatured protein molecules [7,42,65–71]. No intermediate states between native protein forms and start aggregates were detected. Another situation is observed for thermal aggregation of BSA. In this case the average value of the hydrodynamic radius of protein aggregates increases monotonously in time. Hence it is improbable to suggest that start aggregates may form under these conditions. It is worth noting that the kinetic mechanism of BSA aggregation proposed by Sahin et al. [33] does not contain the stages of the formation of nuclei and clusters of nuclei (of the type of start aggregates).

## Supporting Information

S1 Fig SEC elution profile (Sephacryl S100 HR, flow rate of 2.5 ml/min, 20°C) for non-aggregated BSA obtained after heating for 12 h at 60°C. Inset shows the calibration plot.
(PDF)

S2 Fig Analytical ultracentrifugation of SEC-obtained fractions of BSA preheated for 12 h at 60°C. (A) The *c*(*s*) distribution for the fraction eluted in the interval from 82 to 94.6 min. (B) The *c*(*s*) distribution for the fraction eluted in the interval from 94.6 to 122 min. BSA concentration was 0.17 mg/ml for both fractions. Rotor speed was 52000 rpm.
(PDF)

S3 Fig Distribution of the particles by size obtained by DLS. (A) Intact BSA (0.15 mg/ml). (B) Non-aggregated unfolded BSA (the fraction obtained by SEC of BSA preheated for 12 h at 60°C with elution time in the interval from 94.6 to 122 min). BSA concentration was 0.15 mg/ml.
(PDF)

S4 Fig Tryptophan fluorescence spectra for intact BSA (dotted line) and non-aggregated unfolded BSA (solid curve). BSA concentration was 0.1 mg/ml. Excitation was at 298 nm. Conditions: 0.1 M Na-phosphate buffer, pH 7.0, 23°C.
(PDF)

S5 Fig Fluorescence spectra of free ANS (10 μM; curve 1), ANS (10 μM) in the presence of intact BSA (0.1 mg/ml; curve 2) and ANS (10 μM) in the presence of non-aggregated unfolded BSA (0.1 mg/ml; curve 3). Excitation was at 380 nm. Conditions: 0.1 M Na-phosphate buffer, pH 7.0, 23°C.
(PDF)

S6 Fig CD spectra for intact BSA (dotted line) and non-aggregated unfolded BSA (solid curve). BSA concentration was 0.1 mg/ml.
(PDF)

S1 Table Secondary structure content calculated from CD spectra for native BSA and BSA preheated for 12 h at 60°C. (PDF)
