# Supplementary material for: Kinetics of Thermal Denaturation and Aggregation of Bovine Serum Albumin
Source: PLoS One. 2016 Apr 21;11(4):e0153495. doi: 10.1371/journal.pone.0153495 (PMC4839713; doi:10.1371/journal.pone.0153495)
Supplement: S1 Fig — Inset shows the calibration plot. (PDF) [file pone.0153495.s001.pdf]

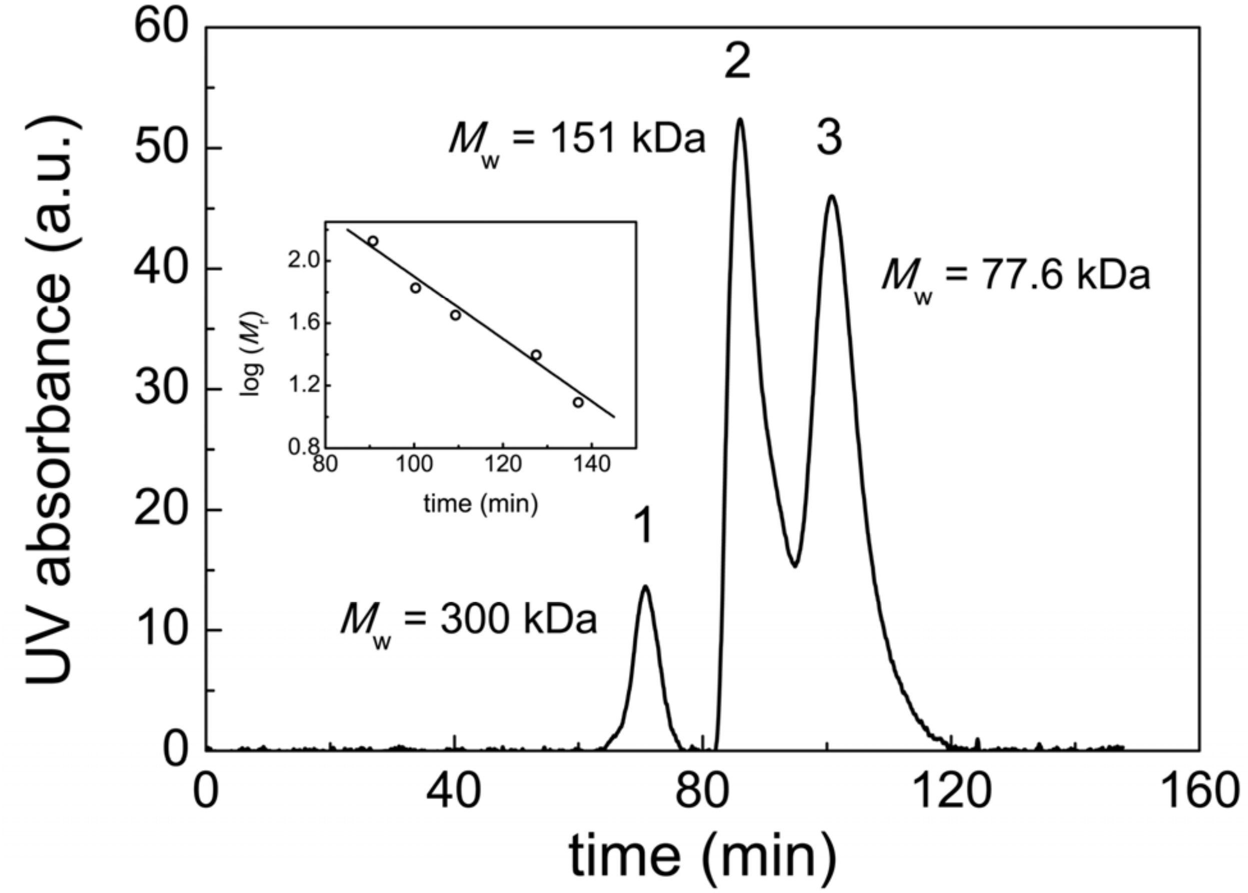

S1 Fig. SEC elution profile (Sephacryl S100 HR, flow rate of 2.5 ml/min, 20 °C) for non-aggregated BSA obtained after heating for 12 h at 60 °C.
