# Supplementary figures and images for: Kinetics of Thermal Denaturation and Aggregation of Bovine Serum Albumin
Source: PLoS One. 2016 Apr 21;11(4):e0153495. doi: 10.1371/journal.pone.0153495 (PMC4839713; doi:10.1371/journal.pone.0153495)

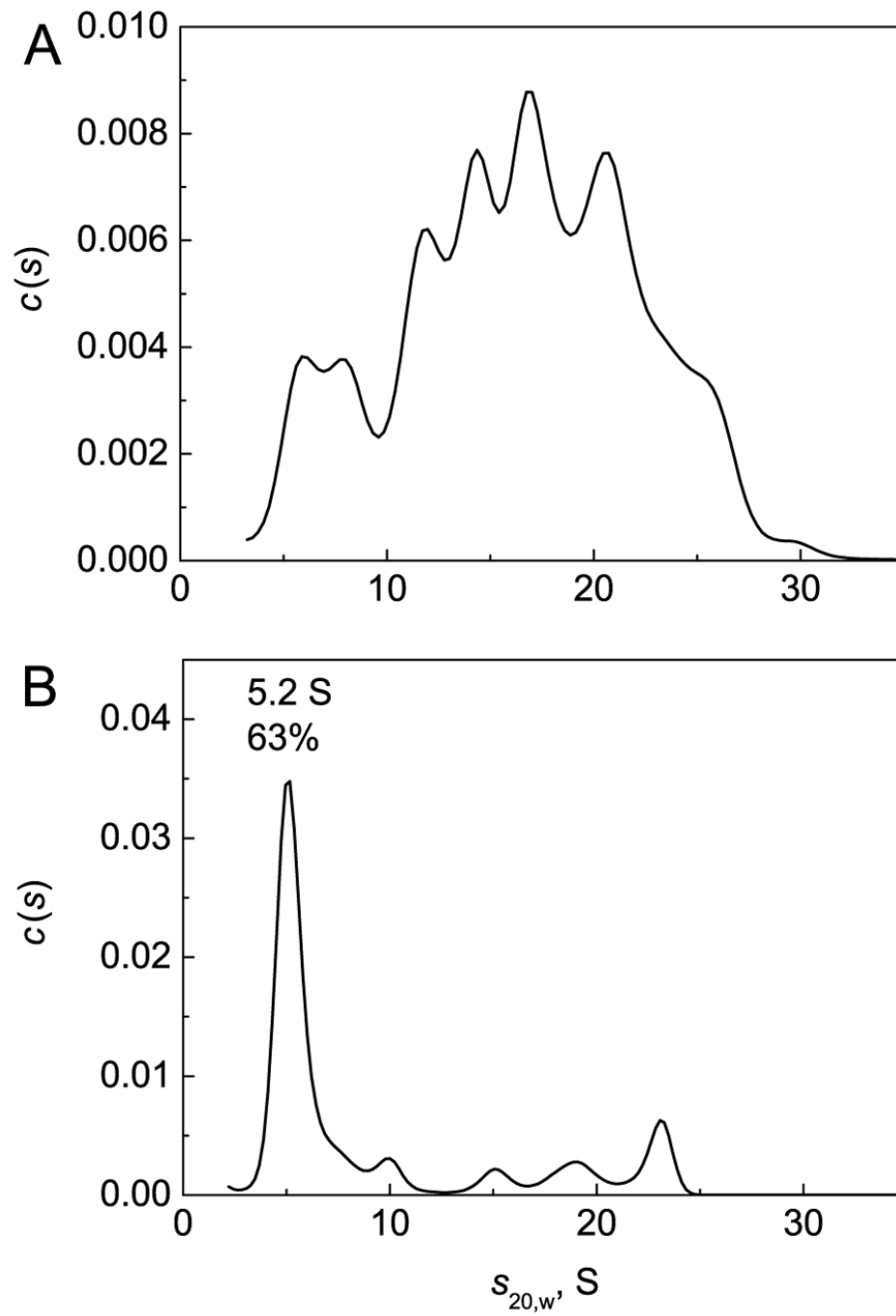

**S2 Fig. Analytical ultracentrifugation of SEC-obtained fractions of BSA preheated for 12 h at 60 °C.**

Supplement: S2 Fig — (A) The c(s) distribution for the fraction eluted in the interval from 82 to 94.6 min. (B) The c(s) distribution for the fraction eluted in the interval from 94.6 to 122 min. BSA concentration was 0.17 mg/ml for both fractions. Rotor speed was 52000 rpm. (PDF) [file pone.0153495.s002.pdf]

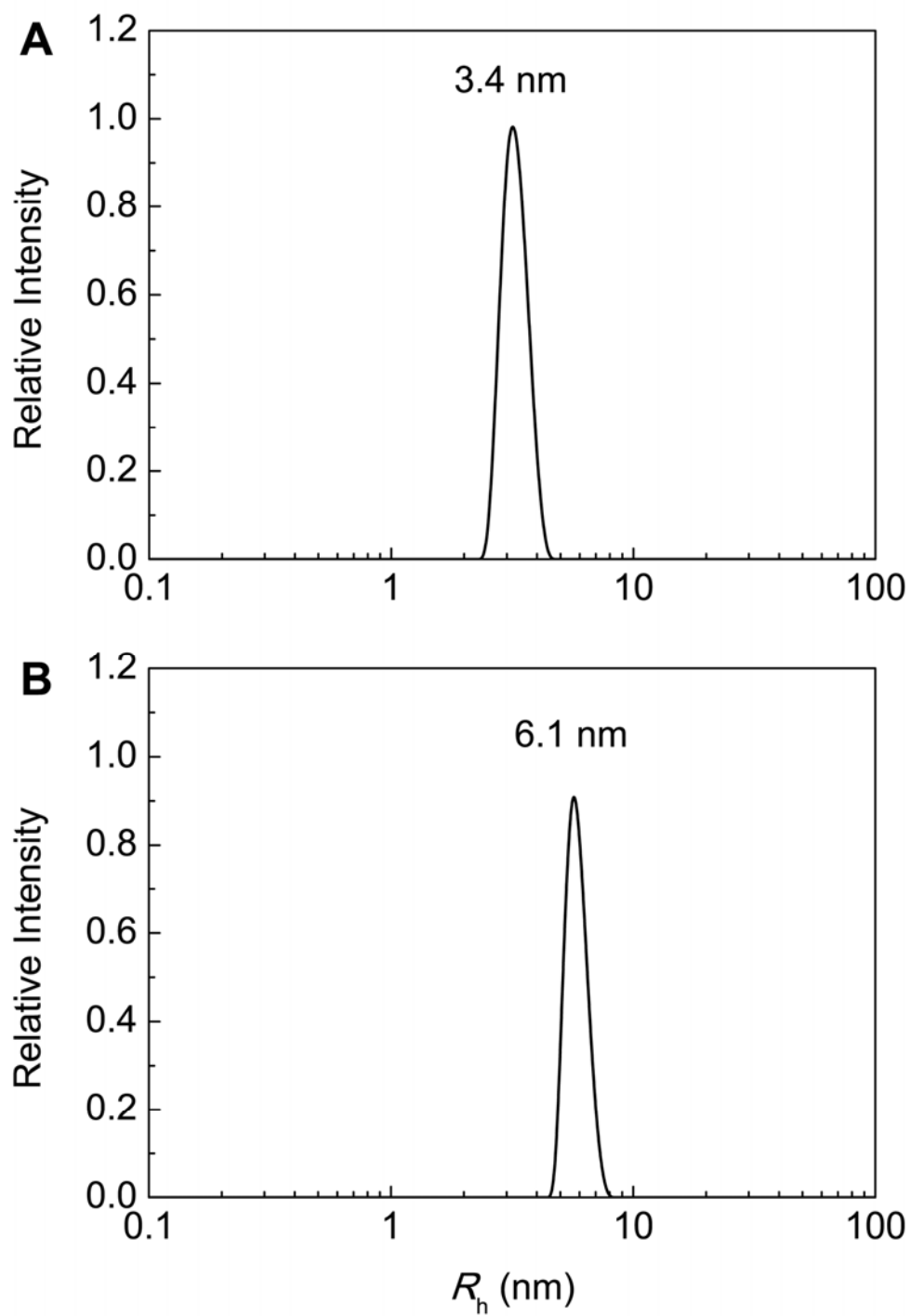

**S3 Fig. Distribution of the particles by size obtained by DLS.**

Supplement: S3 Fig — (A) Intact BSA (0.15 mg/ml). (B) Non-aggregated unfolded BSA (the fraction obtained by SEC of BSA preheated for 12 h at 60°C with elution time in the interval from 94.6 to 122 min). BSA concentration was 0.15 mg/ml. (PDF) [file pone.0153495.s003.pdf]

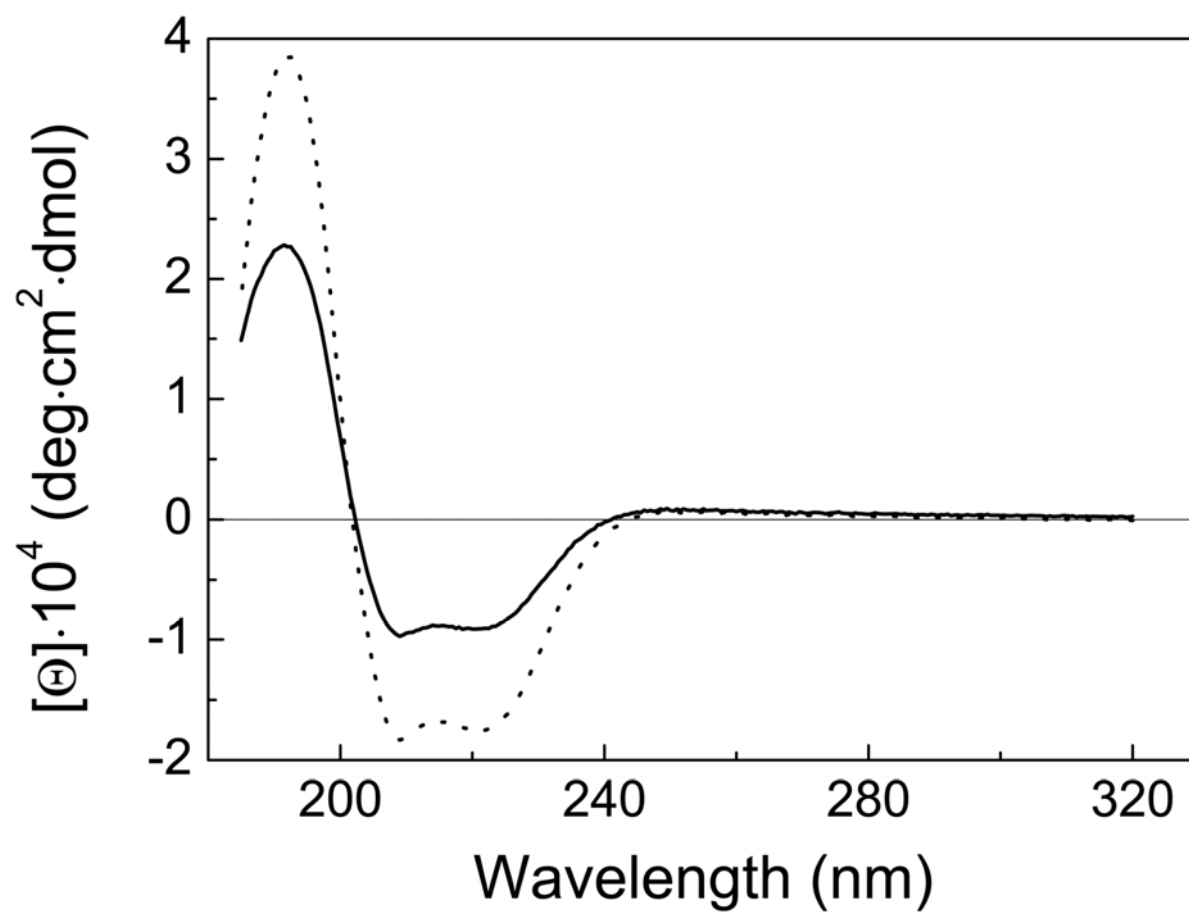

S6 Fig. CD spectra for intact BSA (dotted line) and non-aggregated unfolded BSA (solid curve).

Supplement: S6 Fig — BSA concentration was 0.1 mg/ml. (PDF) [file pone.0153495.s006.pdf]
