# Supplementary material for: Kinetics of Thermal Denaturation and Aggregation of Bovine Serum Albumin
Source: PLoS One. 2016 Apr 21;11(4):e0153495. doi: 10.1371/journal.pone.0153495 (PMC4839713; doi:10.1371/journal.pone.0153495)
Supplement: S4 Fig — BSA concentration was 0.1 mg/ml. Excitation was at 298 nm. Conditions: 0.1 M Na-phosphate buffer, pH 7.0, 23°C. (PDF) [file pone.0153495.s004.pdf]

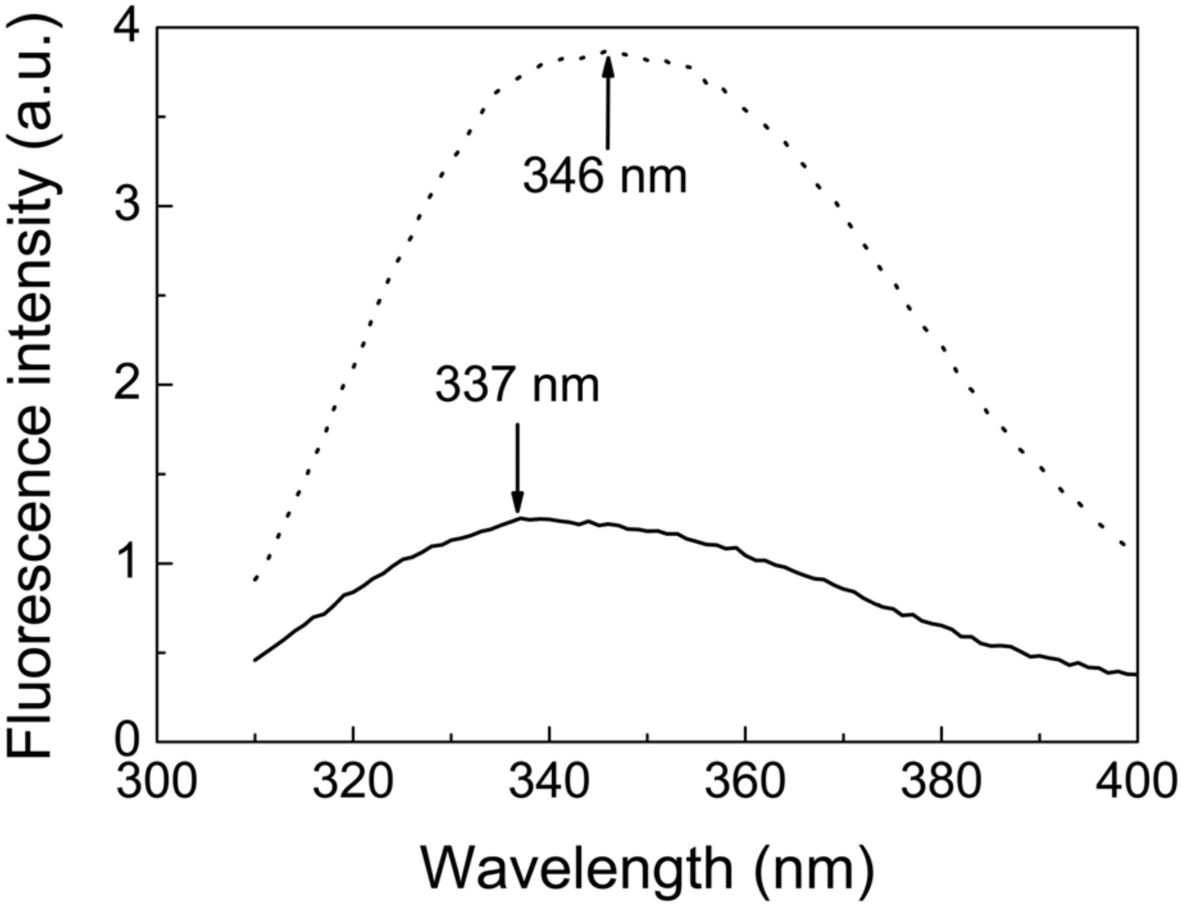

S4 Fig. Tryptophan fluorescence spectra for intact BSA (dotted line) and non-aggregated unfolded BSA (solid curve).
