# Supplementary material for: Kinetics of Thermal Denaturation and Aggregation of Bovine Serum Albumin
Source: PLoS One. 2016 Apr 21;11(4):e0153495. doi: 10.1371/journal.pone.0153495 (PMC4839713; doi:10.1371/journal.pone.0153495)
Supplement: S5 Fig — Excitation was at 380 nm. Conditions: 0.1 M Na-phosphate buffer, pH 7.0, 23°C. (PDF) [file pone.0153495.s005.pdf]

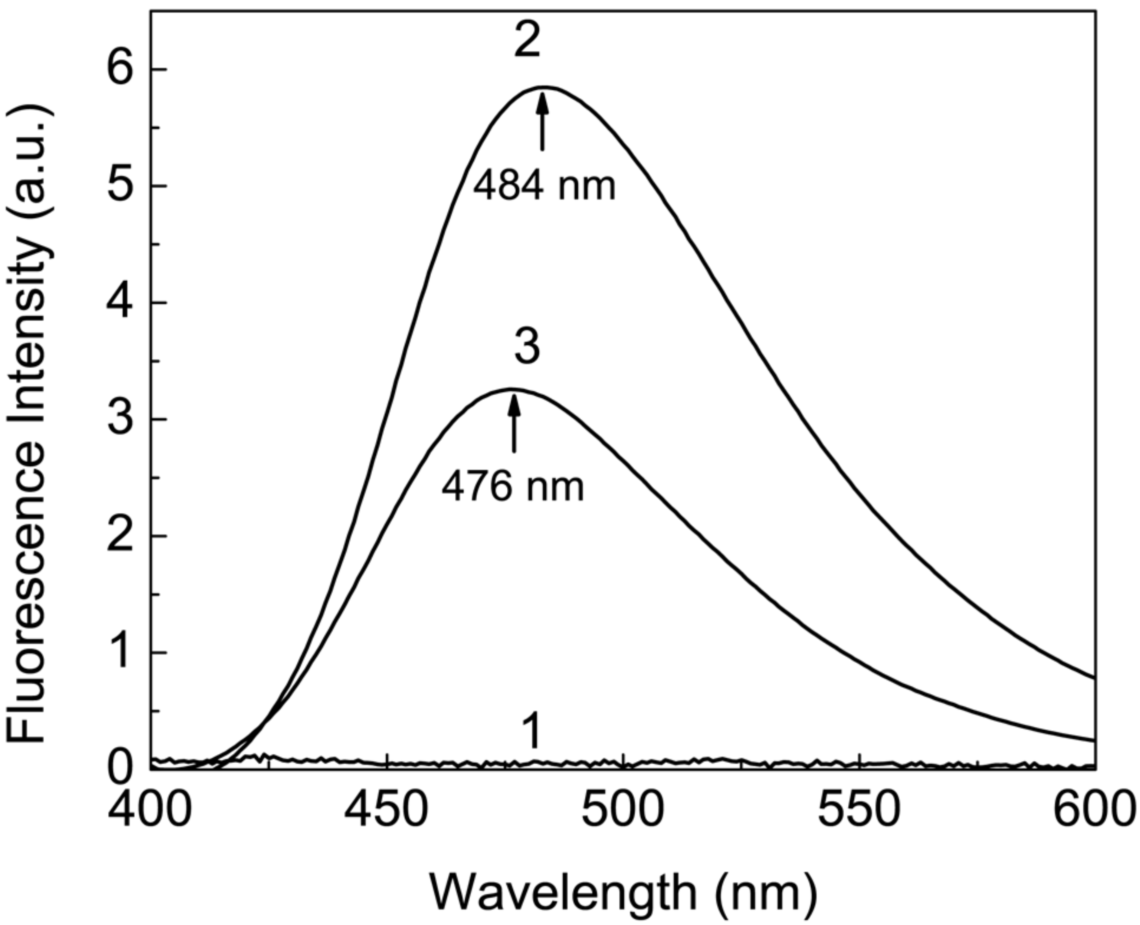

S5 Fig. Fluorescence spectra of free ANS (10 μM; curve 1), ANS (10 μM) in the presence of intact BSA (0.1 mg/ml; curve 2) and ANS (10 μM) in the presence of non-aggregated unfolded BSA (0.1 mg/ml; curve 3).
