# Supplementary material for: Kinetics of Thermal Denaturation and Aggregation of Bovine Serum Albumin
Source: PLoS One. 2016 Apr 21;11(4):e0153495. doi: 10.1371/journal.pone.0153495 (PMC4839713; doi:10.1371/journal.pone.0153495)
Supplement: S1 Table — (PDF) [file pone.0153495.s007.pdf]

S1 Table. Secondary structure content calculated from CD spectra for native BSA and BSA preheated for 12 h.

Secondary structure element	α-Helix	β-Strand	Turns	Unordered
**CONTIN, Set 3**				
**Native BSA**	0.49	0.11	0.16	0.25
**Unfolded non-aggregated BSA**	0.31	0.14	0.18	0.31
**SELCON, Set 3**				
**Native BSA**	0.49	0.10	0.16	0.25
**Unfolded non-aggregated BSA**	0.38	0.16	0.20	0.28
